# Diffusing capacity for carbon monoxide is significantly associated with cardiovascular disease-related plasma proteins, independently of obstruction

**DOI:** 10.1186/s12014-026-09584-6

**Published:** 2026-02-01

**Authors:** Suneela Zaigham, Xingwu Zhou, Magnus Dencker, Sophia Frantz, Morten Kraen, Per Wollmer, Andrei Malinovschi

**Affiliations:** 1 Department of Medical Sciences, Clinical Physiology, Uppsala University, Uppsala, Sweden; 2 Department of Clinical Sciences Malmö, Lund University, Malmö, Sweden; 3 Department of Translational Medicine, Lund University, Malmö, Sweden; 4 Department of research, development, education and innovation, HTA syd, Skåne University Hospital, Lund, Sweden; 5 Department of Medical Sciences, Respiratory, Allergy and Sleep Research, Uppsala University, Uppsala, Sweden

## Abstract

**Background:**

There are known associations between cardiovascular disease (CVD)-related plasma proteins and spirometry measures. Diffusing capacity for carbon monoxide (D_LCO_) measures gas exchange that can be impaired both by lung and heart diseases. We aimed to study the associations between D_LCO_ and CVD-linked plasma proteins in a population-based cohort without airflow obstruction.

**Methods:**

89 CVD-linked proteins were analysed in 427 individuals who underwent spirometry examination with D_LCO_ measurement. Analyses were adjusted for age, gender, height, weight, smoking status and pack years, plates, storage time and cardiovascular morbidity (carotid plaques, hypertension and cardiac medication). Furthermore, a sensitivity analysis (*n* = 362) was carried out after excluding subjects with an FEV_1_/VC ratio < the lower limit of normal (LLN) and steps were taken to ensure a false discovery rate under 5%.

**Results:**

We found 18 proteins negatively associated with D_LCO_%predicted after full adjustments (GLI reference equations). Eleven of these proteins (Fibroblast growth factor 23 (estimated coefficients, (adjusted p-value)): -0.010 (< 0.001), Matrix metalloproteinase-12; -0.011 (< 0.001), Growth differentiation factor 15: -0.008 (< 0.001), C-C motif chemokine 20: -0.013 (0.006), Interleukin-6: -0.014 (< 0.001), Fatty acid-binding protein, adipocyte − 0.007 (0.001), Urokinase-type plasminogen activator receptor: -0.004 (< 0.001), Interleukin-1 receptor antagonist: -0.008 (< 0.001), TNF-related apoptosis-inducing ligand receptor 2: -0.005 (0.001), Renin: -0.008 (0.03) and Spondin-1: -0.003 (0.03)) remained significant after further excluding subjects with obstruction on spirometry (FEV_1_/VC < LLN).

**Conclusions:**

Several CVD-linked plasma proteins were associated with D_LCO_ in subjects without airflow obstruction on spirometry and after adjustments for known cardiovascular morbidity. The likely explanations may be the pro-fibrotic and pro-inflammatory nature of many of the proteins causing changes in gas exchange. These proteins could potentially signal for early disease mechanisms leading to impaired gas exchange.

**Supplementary Information:**

The online version contains supplementary material available at 10.1186/s12014-026-09584-6.

## Key messages

- Cardiovascular disease is associated with low lung function. To establish the underlying mechanisms studies have found a link between CVD-associated plasma proteins and FEV_1_ and FVC but no population-based proteomic studies assessing D_LCO_ have been carried out.
- We found several CVD-associated plasma proteins related to D_LCO_, independent of obstruction on spirometry, many of which are linked to profibrotic or proinflammatory processes.
- The mechanisms of the significant proteins can give us a better understanding of the potential pathophysiological processes that link D_LCO_ to CVD outcomes.

**Supplementary Information:**

The online version contains supplementary material available at 10.1186/s12014-026-09584-6.

## Introduction

There is a long known and well-established link between low lung function and cardiovascular disease (CVD) that has been consistently reported in observational studies. This finding has been confirmed in genetic studies, where certain spirometry findings are causally linked to cardiovascular events such as coronary heart disease [1]. The mechanisms behind this strong association remain unclear, which has prompted studies assessing the proteome, specifically assessing CVD-linked plasma proteins and if any associations with low lung function can indicate pathways that may explain this relationship.

Some studies have now found significant associations between measures of spirometry and CVD-linked plasma proteins. We have previously reported proteins associated with lung volumes and not obstruction on spirometry [2]. The Framingham heart study also assessed the link between spirometry and CVD-linked plasma proteins in a large community-based cohort and found significant associations between forced expiratory volume in 1 s (FEV_1_) along with forced vital capacity (FVC), but not the ratio of the two with plasma proteins, including markers of inflammation, adiposity and fibrosis [3]. Similarly, other studies have found significant associations between plasma proteins such as GDF-15 and reduced FEV_1_ and even found this link to be causal [4]. Previous studies have also assessed common proteomic biomarkers in pulmonary and coronary artery disease in order to better understand the link between COPD and coronary artery disease (CAD) [5]. The Swedish Biomarkers and Genetics CardioPulmonary Physiology Study (BiG CaPPS) found subjects with CAD had a lower D_LCO_ than those without CAD [5]. In the study although common proteomic biomarkers of chronic airflow obstruction and CAD were assessed, airflow obstruction was defined according to spirometry alone [5]. Therefore, the majority of the existing important literature on this subject assesses spirometry measures as the measure of lung function.

Diffusing capacity for carbon monoxide (D_LCO_) assesses the lungs’ ability to transfer gas from the air into the bloodstream. It can give important information on the nature of respiratory pathology that may be present and can also potentially provide an early indication of disease that may not yet be measurable by spirometry, such as in emphysema, interstitial pulmonary fibrosis or any interstitial lung disease [6]. Although there have been some general population studies that have not found gas exchange to be linked to CVD risk [7, 8], D_LCO_ has a well-documented link to heart failure [9] and has been linked to plaques in the internal carotid artery in the general population, independently of established atherosclerotic risk factors, suggesting the relationship between lung function, COPD and CVD are not only related to bronchial disease and obstruction or low grade systemic inflammation [10]. Proteomic studies related to D_LCO_ can therefore aid in identifying such additional mechanisms.

There have been studies assessing the association between plasma proteins and D_LCO_ in specific patient groups such as COPD [11] and idiopathic pulmonary fibrosis [12], however, studies assessing the link between CVD-linked plasma proteins and D_LCO_ in the general population are lacking. The Role of Low Lung function Study (ROLLS) on which the present study is based has previously found FGF-23 [13] and matrix metalloproteinases (MMPs) [14] to be significantly associated with D_LCO_ along with other pulmonary function measures, however the association of further CVD-linked plasma proteins with a focus on D_LCO_ needs further exploring to identify specific pathways related to CVD.

We aim to assess the association between CVD-linked plasma proteins and D_LCO_ in the general population and if any associations are independent of pulmonary obstruction and cardiovascular comorbidity.

## Methods

### Study population

The study population consists of the ROLLS cohort carried out at the Department of Medical Imaging and Physiology, Skåne University Hospital, Malmö, Sweden between 15th June 2004- 10th May 2007. The study population has been described in detail elsewhere [15, 16]. Briefly, it is a sub-population of participants from a postal respiratory survey (“Question about the lungs”) which was performed in a randomly selected adult population in southern Sweden in the year 2000 (*n* = 11 933). For the ROLLS study [10], 870 subjects from that population who were living in Malmö were invited. Selection of ROLLS was stratified for smoking habits and self-reported COPD. Of the 870 invited, 450 subjects took part in the study. Biomarkers were analysed in 2015. The study was approved by the Ethics committee of Lund University (LU 786-03 and 2015/201). All participants in the study signed an informed written consent before participation. All methods were performed in accordance with the relevant guidelines and regulations. Patients or the public were not involved in the design, or conduct, or reporting, or dissemination plans of our research.

### Questionnaire response

The postal respiratory survey was used both as part of the study recruitment and for classification of subjects at study visit. The questionnaire included questions on smoking status and respiratory symptoms. Subjects who had never smoked daily for more than one month were classified as never smokers. Current and ex-smokers were classified as ever-smokers. Total tobacco consumption was calculated in pack years (one pack year = 20 cigarettes smoked/day for one year).

### Lung function measurement

All lung function measurements were taken 15 min after inhalation of 1.0 mg of Terbutaline. Spirometry was performed according to ERS recommendations [17]. A spirometer (Master Screen; Viasys GmbH – Erich Jaeger, Hoechberg, Germany) was used to measure FEV_1_ and vital capacity (VC). D_LCO_ was measured using the single-breath technique according to Cotes et al. [18]. The reference values for D_LCO_ were corrected for haemoglobin values according to established procedures [19]. D_LCO_%predicted values and D_LCO_/alveolar volume _(VA)_%predicted values were calculated according to the Global Lung Initiative reference equations (GLI) [20]. Obstruction was defined as FEV_1_/VC < the lower limit of normal (LLN) using GLI equations.

### Proteomics

The Proseek Multiplex CVD I 96 × 96 assay (Olink, Bioscience, Uppsala, Sweden) measuring 92 CVD-related human proteins with the proximity extension assay method was used in this study [21, 22]. In the present study, 89 proteins across five plates were available for analysis (three proteins were excluded due to a high proportion [more than 15%] of values below the limit of detection (LOD). For the remaining proteins, values below the LODs were imputed using the corresponding LOD/sqrt [2]. Blood samples were collected between 2004 and 2007 and stored at − 80 °C until 2016, when the analyses were performed.

### Cardiovascular co-morbidity

Cardiovascular comorbidity was assessed three ways (a) ultrasonography of atherosclerotic plaques in the internal carotid artery (ICA) (b) blood pressure measurement (systolic BP > 140 mmHg OR diastolic BP > 90mmHg and c) use of cardiac medication. The presence of any of the three indicated cardiac morbidity. A 7.5 MHz linear ultrasound probe was used to screen for plaques in the ICA, bulb and distal portion of the common carotid artery bilaterally for plaques (absent or present). A present plaque was defined as focal thickening of the total vessel wall (≥ 2 mm) relative to adjacent segments, protruding into the lumen in at least one side. Blood pressure was measured to the nearest 5 mmHg after 5–10 min of rest in the supine position, in the right upper arm. The mean of two measurements was calculated. Cardiac medication was self-reported use of cardiovascular medication with the question “Do you take any medicine for heart disease (e.g. heart failure, angina) or for high blood pressure? If yes, which medications and when?”

### Statistical analysis

Continuous variables are presented as mean (standard deviation, SD) andcategorial variables are presented as numbers (proportions, %).

From 450 subjects who initially took part in the study, 23 subjects were excluded due to missing data on proteomics/technical or quality control issues in the sample (*n* = 22) or other covariates (*n* = 1). Supplement Fig. 1 shows the flow of subjects through the study. A total of 427 subjects, 89 proteins on 5 plates were analysed.

**Fig. 1:**
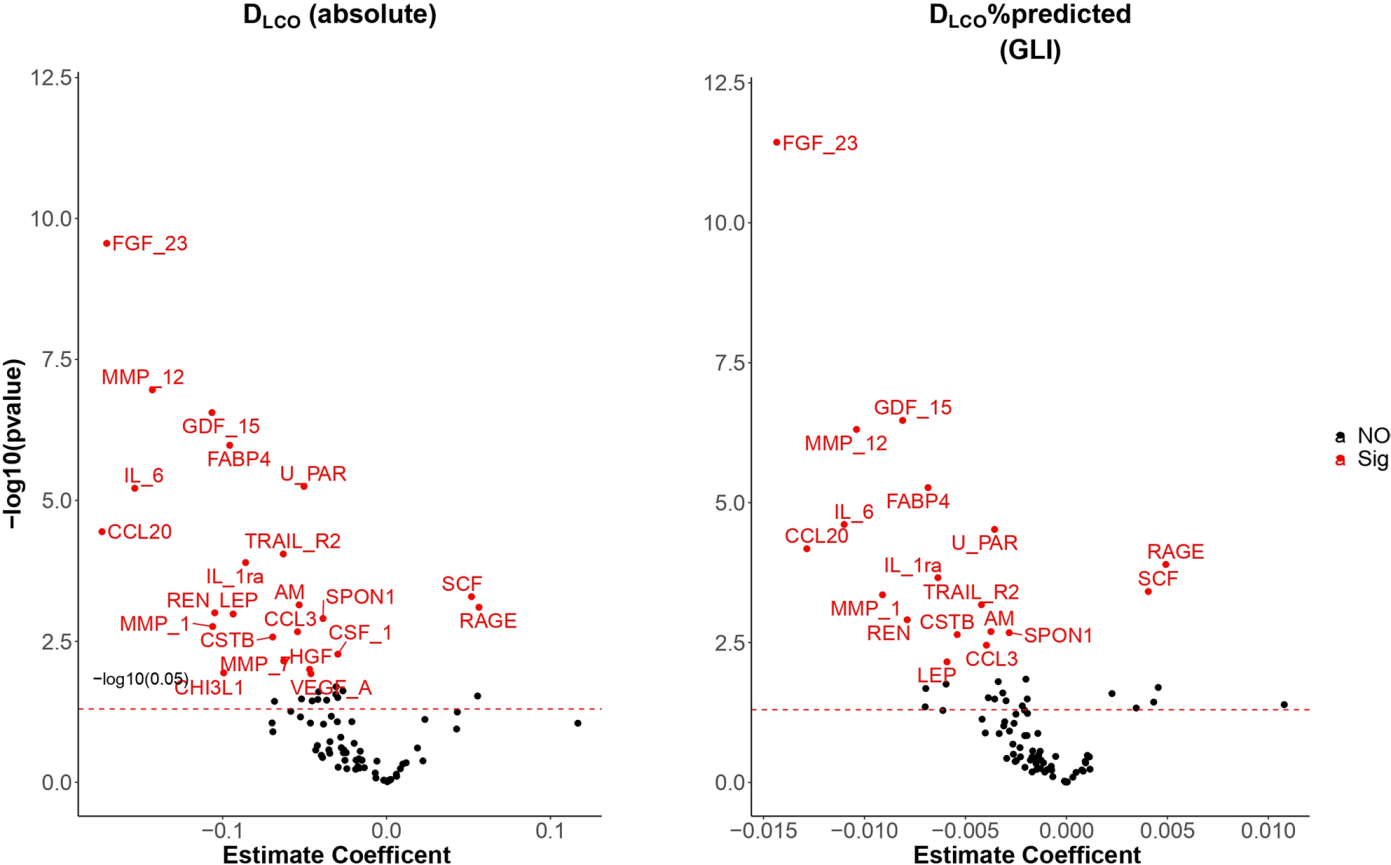
Significant proteins associated with D_LCO_ (absolute) – panel 1, D_LCO_%pred (GLI equations)- panel 2. (Adjusted for the basic confounders and for CVD morbidity)

Multiple linear regression models were implemented to assess the association between proteins and the D_LCO_ measures. D_LCO_ measures (D_LCO_, D_LCO_%predicted (D_LCO_%pred) and D_LCO_/V_A_ %predicted) were used as the independent variables and proteins were used as the dependent variables.

All analyses were adjusted for age, gender, height, weight, smoking status and pack years, plate and storage time (Model 1). An additional model was carried out where further adjustments for cardiovascular morbidity was made (carotid plaques, hypertension and cardiac medication) (Model 2). A sensitivity analysis was carried out for Model 2 after excluding subjects with obstruction on spirometry (FEV_1_/VC ratio of < LLN). All analyses were adjusted for plate and storage time. A p-value < 0.05 was considered statistically significant.

All analyses were performed using R version 4.5.2 (R Core Team) [23].

## Results

Baseline characteristics are shown in Table 1. Mean age of the subjects was 61.5 years and almost half of the cohort were current smokers (49.2%). Over half of the cohort had a history of carotid plaques (51.5%) and a large proportion of the cohort had CVD as defined by the presence of either carotid plaques, hypertension or taking cardiac medication (70.4%). There was a 16% and 14% prevalence of self-reported COPD in the main cohort and cohort after excluding obstruction on spirometry, respectively.

**Table 1:** Baseline characteristics

	Main analysis (*N* = 427)	Sensitivity analysis (*N* = 362)
Age (years)	61.5 (7.6)	61.1 (7.7)
Sex (n, % female)	250 (58.5)	213 (58.8)
Height (cm)	169.5 (8.9)	169.2 (8.8)
BMI (kg/m^2^)	26.6 (5.0)	26.8 (5.1)
Current smokers (n, %)	210 (49.2)	175 (46.0)
Never smokers (n, %)	82 (19.3)	79 (25.0)
Carotid plaques (n, %)	219 (51.5)	177 (49.0)
Cardiac medication (n, %)	99 (23.2)	77 (21.3)
Hypertension (n, %) (Systolic BP > 140 or Diastolic BP > 90)	164 (38.4)	132 (36.5)
* Cardiovascular diseases (n, %)	300 (70.4)	248 (68.7)
Systolic blood pressure (mmHg)	138.2 (18.3)	137.3 (17.5)
Diastolic blood pressure (mmHg)	77.8 (11.2)	77.2 (10.8)
Self-Reported COPD (n, %)	67 (16.1)	50 (14.2)
D_LCO_ (mmol min^− 1^ kPa^− 1^)	6.9 (2.0)	7.1 (1.9)
D_LCO_ (%predicted, GLI)	89.9 (18.9)	92.5 (17.4)
FEV_1_ (L)	2.7 (0.8)	2.8 (0.7)
FEV_1_/VC	0.74 (0.09)	0.77 (0.06)
V_A_ < LLN (GLI) (n, %)	38 (9.0)	30 (8.4)

Data are mean (SD) unless otherwise stated. * Cardiovascular disease variable composed of yes to any of the following three: Carotid plaques, hypertension (systolic > 140 or diastolic > 90), cardiac medication). COPD: Chronic Obstructive Pulmonary Disease (self-reported). LLN: lower limit of normal, GLI: Global lung initiative

In the cohort of 427 subjects, 17 proteins were associated significantly with D_LCO_%pred after adjusting for Model 1 controlled at an FDR less than 0.05 (Table 2).

**Table 2:** D_LCO_%pred associations with proteins

Significant proteins in whole cohort (*n* = 427) (Model 1)	Significant proteins in whole cohort (*n* = 427) (Model 2)	Significant proteins after excluding FEV_1_/VC < LLN (*n* = 362) (Model 2)
FGF-23 MMP-12 GDF-15 CCL20 IL-6 FABP4 IL-1ra TRAIL-R2 U-PAR MMP-1 AM CSTB CCL3 RAGE (^+^) REN SCF (^+^) SPON1	FGF-23 MMP-12 GDF-15 CCL20 IL-6 FABP4 IL-1ra TRAIL-R2 U-PAR MMP-1 AM CSTB CCL3 RAGE (^+^) REN LEP SCF (^+^) SPON1	FGF-23 MMP-12 GDF-15 CCL20 IL-6 FABP4 IL-1ra TRAIL-R2 U-PAR REN SPON1

Model 1 age, gender, height, weight, smoking status, pack years, plate, and storage time

Model 2 age, gender, height, weight, smoking status, pack years, plate, storage time, CVD

All proteins are negatively associated with D_LCO_%pred unless otherwise stated with the (^+^) notation, where it represents a positive association

After further adjusting for cardiovascular morbidity (Model 2), 18 proteins were associated with D_LCO_%pred (addition of leptin) (Table 2 and Table 3). After further excluding subjects with obstruction on spirometry **(**Model 2 sensitivity analysis), 11 proteins remained associated (Tables 2 and 3). These 11 proteins (GDF-15, IL-1ra, IL-6, FGF-23, MMP-12, CCL-20, FABP4, U-PAR, TRAIL-R2, REN and SPON1) were also significantly associated with absolute values of D_LCO_, Model 2 sensitivity analyses, with the addition of Leptin (12 proteins) (see Supplement material for full D_LCO_ results).

**Table 3:** Significant associations between D_LCO_%pred and plasma proteins (Model 2 sensitivity analysis)

Protein	Abbreviation	Estimate (standard error)	Adjusted *P*-value
Fibroblast growth factor 23	FGF-23	−0.010 (0.002)	< 0.001
Macrophage metalloelastase 12	MMP-12	−0.011 (0.002)	< 0.001
Growth/differentiation factor 15	GDF-15	−0.008 (0.002)	< 0.001
C-C motif chemokine 20	CCL20	−0.013 (0.004)	0.006
Interleukin-6	IL-6	−0.014 (0.003)	< 0.001
Fatty acid-binding protein, adipocyte	FABP4	−0.007 (0.002)	0.001
Urokinase-type plasminogen activator receptor	U-PAR	−0.004 (0.001)	< 0.001
Interleukin-1 receptor antagonist	IL-1ra	−0.008 (0.002)	< 0.001
TNF-related apoptosis-inducing ligand receptor 2	TRAIL-R2	−0.005 (0.001)	0.001
Renin	REN	−0.008 (0.003)	0.030
Spondin-1	SPON1	−0.003 (0.001)	0.030

Adjusted for age, gender, height, weight, smoking status and pack years, plate, storage time, CVD. Those with obstruction on spirometry (FEV_1_/VC ratio < LLN) have been excluded as part of the sensitivity analysis

The relationship between the 11 significant proteins from the sensitivity analysis and D_LCO_%pred values is displayed in Supplement Fig. 3. The relationships were found to be linear across the range for all proteins.

In the sensitivity analyses, IL-6 was associated with the largest negative estimate for D_LCO_%pred (estimate (standard error): −0.014 (0.003), *p* < 0.001), followed by CCL20 (−0.013 (0.004), p-0.006). MMP-12 and FGF-23 were also associated with similar negative estimates for D_LCO_%pred (−0.011 (0.002), *p* < 0.001 and − 0.010 (0.002), *p* < 0.001, respectively).

All proteins found to be significant in the sensitivity analyses, with the exception of three (IL-1ra, FABP4 and SPON1) had higher levels for D_LCO_< LLN vs. > LLN, *p* < 0.05 (according to GLI equations) (Supplement Fig. 2).

**Fig. 2:**
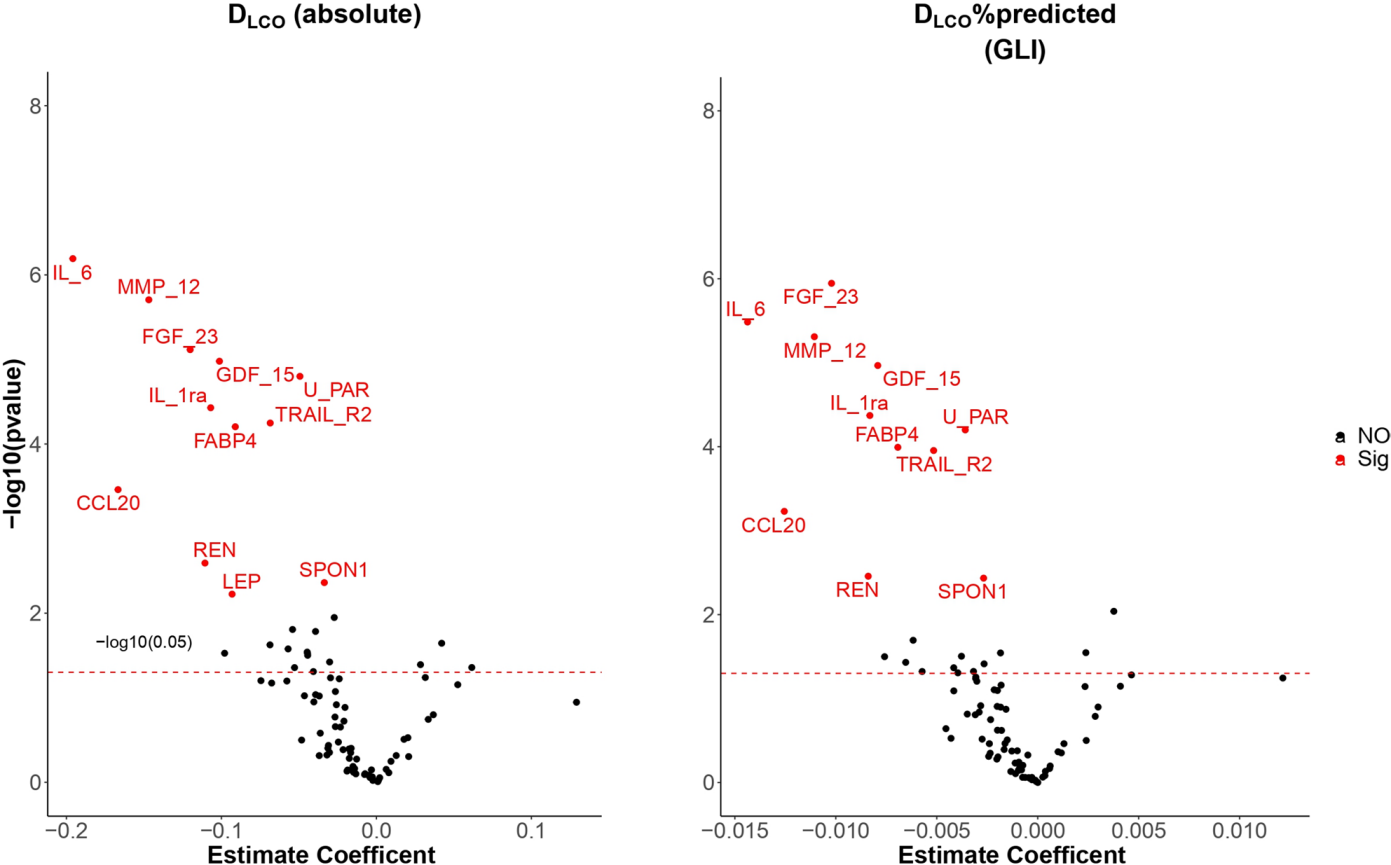
Significant proteins associated with D_LCO_ (absolute) – panel 1, D_LCO_%pred (GLI equations)- panel 2. Adjusted for the basic confounders and for CVD morbidity and subjects with FEV_1_/VC < LLN excluded)

After further excluding subjects with self-reported COPD from the sensitivity analysis, we found the same 11 proteins remained significantly associated with D_LCO_%pred, with the addition of two more proteins, Leptin and MMP-1 (estimates (standard error): −0.01(0.003), FDR adjusted p-value, 0.01 and − 0.01 (0.004), FDR adjusted p-value 0.0497, respectively) (Results not shown elsewhere).

Figure 1 shows significant proteins associated with D_LCO_ (absolute) – panel 1, D_LCO_%pred (GLI equations)- panel 2. The results are adjusted for the basic confounders and for CVD morbidity (Model 2). Figure 2 shows significant proteins associated with D_LCO_ (absolute) – panel 1, D_LCO_%pred (GLI equations)- panel 2. The results are adjusted for the basic confounders and for CVD morbidity (Model 2) and also exclude subjects with FEV_1_/VC < LLN (Model 2 sensitivity analysis).

### Associations between proteins and D_LCO_/V_A_%pred

In 418 subjects, the association between D_LCO_/V_A_%pred was tested for the final models: Model 2 and Model 2 sensitivity analysis excluding obstruction on spirometry. Two proteins were significantly associated with D_LCO_/V_A_%pred, (FGF-23 (estimated coefficient − 0.012, adjusted p<0.001), MMP-12 (−0.009, adjusted p-0.006). After excluding those with obstruction on spirometry, no proteins remained significantly associated with D_LCO_/V_A_%pred. However, after excluding subjects with V_A_ (alveolar volume) < LLN there were 5 proteins associated with D_LCO_/V_A_%pred (FGF-23, MMP-12, GDF-15, U-PAR, IL-6RA) and in the sensitivity analysis (excluding obstruction on spirometry) after additionally excluding V_A_< LLN, 8 proteins remained significantly associated with D_LCO_/V_A_%pred (FGF-23, MMP-12, U-PAR, GDF-15, IL-6RA, CCL3, FABP4, CD40) (Supplement material).

### Obstruction on spirometry and association with proteins

We assessed if any proteins were related to obstruction (FEV_1_/VC < LLN using FEV_1_/VC > = LLN as the reference group after adjustment for other covariates. No proteins were identified under FDR less than 0.05.

## Discussion

In this cross-sectional study we found 11 CVD-linked proteins to be associated with D_LCO_, even after adjusting for potential confounders including cardiovascular morbidity and after excluding subjects with obstruction on spirometry.

Low D_LCO_ can either be isolated or found together with airways obstruction, the later often in COPD with emphysematous changes, while the diseases behind isolated D_LCO_ are less studied. Theoretically, interstitial abnormalities, heart failure as well as emphysematous changes can be related to isolated D_LCO_ reduction. These differences together with a reduction of statistical power might explain the fewer associations found in the sensitivity analyses after exclusion of subjects with airways obstruction.

The ROLLS cohort has previously explored the association between plasma proteins and COPD and/or lung function [13, 14, 24], including two of the significant plasma proteins associated with D_LCO_ (FGF-23 and MMP-12) [13, 14] in the present study, and also found significant relationships between these two proteins and D_LCO_. However, these previous studies have differed either in adjustments/exclusions in their linear regression models to the present study [13], or carried out only correlation analyses with regards to proteins and D_LCO_%pred [14]. We additionally also have FDR-corrections in the present study.

Exposure of airway epithelial cells to cigarette smoke and FGF-23 has been found to lead to a significant increase in interleukin-1β release [25] – known to be a crucial inflammatory cytokine in the lung involved in the initiation and persistence of inflammation [26] Meta-analysis of epidemiological studies have found that FGF-23 is associated with a high risk of atherosclerotic cardiovascular disease [27], however, a recent Mendelian randomization study found that genetically predicted FGF-23 levels were not causally linked with atherosclerotic and non-atherosclerotic diseases, suggesting that previously found epidemiological associations may have been observed due to some residual confounding [28]. Therefore, it seems unclear if FGF-23 has a pathophysiological link from low lung function to CVD outcomes.

MMP-12, also known as macrophage metalloelastase has been found to be associated with rapid lung function decline, and is known to have proteolytic activity causing tissue destruction and elastin degradation [29], thought to be one of the pathophysiological mechanisms of its action in COPD. MMP-12 expression in human atherosclerotic plaques is increased > 300-fold in comparison to atherosclerotic free arteries [30, 31]. Studies assessing D_LCO_ decline in relation to MMP-12 are scarce, however previously the ROLLS cohort has found MMP-12 was a predictor of concomitant COPD and carotid plaques [14], which also supports the hypothesis that MMP-12 and its elastin degradation properties explain part of the associations between D_LCO_ and atherosclerotic diseases.

IL-1ra is an inhibitor of the inflammatory activities of IL-1 – which has a well-documented role in atherosclerosis, acute myocardial infarction and heart failure [32]. Low IL-1ra levels have been found in asthma and COPD [33, 34] therefore one would also expect lower IL-1ra levels with lower levels of D_LCO_. However, we found a significant negative association between D_LCO_ and IL-1ra. A similar association between spirometry measures and IL-1ra was also found in previous study of ours [2]. Although the reason for this is still unclear, we previously speculated that early on in disease processes when there is a heightened inflammatory state, the body may react with initially increasing IL-1ra levels which may be why we observe a negative rather than positive association [2].

IL-6 is a well-known cytokine related to oxidative stress and inflammation and plays a key role in the pathogenesis of CVD [35]. We found higher D_LCO_ levels to be associated with lower IL-6 levels, which is in line with previous findings of spirometry [2, 36]. IL-6 has also been found to have a pro-fibrotic property and related to interstitial lung diseases [37] potentially due to increased membrane thickness resulting from IL-6 pro-fibrotic activities.

We found higher D_LCO_ levels to be associated with lower levels of GDF-15, which is in line with previous findings where higher GDF-levels have been associated with COPD exacerbations, COPD mortality and decline in FEV_1_ and FVC [38]. Higher levels of GDF-15 levels are also associated with other adverse health outcomes including CVD [39]. It has also been found that pulmonary epithelial cells are the most likely source of GDF-15 in the lungs [40] and that GDF-15 is a useful biomarker of epithelial stress where it can identify patients with idiopathic pulmonary fibrosis that may have poor outcomes [41]. Cigarette smoking is thought to increase GDF-15 expression in airway epithelial cells and is also involved in inducing cellular senescence [40]. Alongside this, GDF-15 is also involved in the process of pulmonary vascular remodelling [40]. It has been suggested that the accumulation of senescent cells within the lungs may potentially be a key factor in the pathogenesis of conditions such as COPD [40].

CCL20 has been found to be one of the proteins significantly associated with self-rated health and could explain the association between self-rated health and CVD [42]. In a study of COPD patients, CCL20 levels were significantly higher in induced sputum of patients with COPD compared to never smokers [43]. Chronic exposure to cigarette smoke is thought to promote CCL20 expression. This in turn increases presence of pulmonary dendritic cells that drive immune responses in the airways [43]. Further, blocking CCL20 appears to reduce the presence of dendritic cells and emphysema in a COPD rat model [44].

FABP4 along with other adipocytokines, are thought to involved in the development of lung diseases [45] and have also been related to atherosclerosis. In apparently healthy subjects with normal lung function, serum FABP4 levels increased as lung function (FEV_1_ or FVC) decreased even after adjustments for many potential confounders [46]. This is in line with our previous findings of spirometry [2] and findings from the present study where we found higher D_LCO_ levels to be significantly associated with lower levels of FABP4. FABP4 is thought to act as a pro-inflammatory adipokine. It is related to low-grade systemic inflammation, related to the secretion of cytokines such as IL-6 and TNF-α [47, 48] both of which are related to airway and alveolar inflammation in lung diseases such as COPD and asthma.

FABP4 is also involved in promoting macrophage activation which within the M1 phenotype has pro-inflammatory actions. This is also relevant for the lung where macrophages are involved in airway remodelling and parenchymal inflammation. Additionally, as D_LCO_ levels are affected by changes at the alveolar-capillary membrane; endothelial cell function and inflammation affected by FABP4 [49] may also play a role in lower D_LCO_ levels related to FABP4.

It is believed that U-PAR promotes inflammatory cell migration and activation, extracellular matrix degradation and MMP activation [50] and has been previously associated with COPD [51]. It may therefore be through these mechanisms that it plays a role in emphysema and subsequently is reflected in lower D_LCO_ levels.

TRAIL-R2 (also known as DR5) is a death inducing receptor for TRAIL and has been implicated in cancer but recent evidence suggests a role in CVD. TRAIL and its receptors including TRAIL-R2 are associated with cardiovascular risk factors including smoking, coronary artery disease and diabetes [52] and have been suggested as potential biomarkers for development and progression of cardiac diseases [52]. TRAIL-R2 and TRAIL levels have also been found to be higher in patients with COPD vs. healthy controls and negatively correlated with lung function [53]. There therefore may be a pro-apoptotic and pro-inflammatory function of TRAIL and its receptors associated with lung function [54].

Renin is an enzyme involved in blood pressure regulation via the renin-angiotensin-aldosterone system (RAAS) with important links to CVD [55]. A significant association has previously been found between FEV_1_ and Renin [4] and it has also been found that the RAAS plays an active role in pathogenesis of fibrotic lung diseases [56], therefore it seems plausible that the RAAS contributes to lung fibrogenesis which may be reflected in alterations in D_LCO_ levels.

SPON1 is a cell adhesion protein and has previously been linked causally to heart failure risk factors and left ventricular ejection fraction [57]. As D_LCO_ is also linked to heart failure is seems probable that SPON1 is one of the proteins involved in lower D_LCO_ levels through its effects on left ventricular function as we did not specifically exclude subjects with heart failure as part of CVD exclusions in this study.

Fewer proteins were related with D_LCO_/V_A_%pred and none after excluding obstruction. However, after excluding subjects with abnormal alveolar volume (V_A_) we found several proteins to be associated with D_LCO_/V_A_%pred. This may indicate relationships to changes at the alveolar-capillary membrane and gas exchange efficiency in presence of normal V_A_ e.g., without concomitant peripheral obstruction/ventilation heterogeneity and/or reduced lung volumes. Some of these proteins were also those identified as being associated with D_LCO_%pred (including FGF-23, MMP-12 and GDF-15), strengthening the evidence for changes at the alveolar-capillary membrane related to these proteins.

A notable limitation of the present study is that we could not find a suitable replication cohort therefore we were not able to validate our findings in a replication study. Although the study is population based, the recruitment of study subjects was based on invitations therefore there is a degree of selection bias that should be considered. However due to the explorative nature of the study, this is probably not affecting the validity of our findings. After excluding subjects with obstruction on spirometry, 14% of this sub-cohort still had reported COPD at study inclusion, therefore the role of obstruction in the sensitivity analysis may not be fully excluded. However, we were able to confirm the main results after further excluding subjects with self-reported COPD. An additional limitation is that our cohort is approximately 20 years old. Risk factors and guidelines change over time and therefore one may speculate that the findings are not applicable to current day. However, lung function was thoroughly assessed and plasma proteins were assessed as part of a widely used protein panel still available at present. Therefore, we think that these results are relevant and that it is unlikely the time of cohort measurements would have impacted our results to a great degree. Our definition of CVD in the analysis can be seen as a limitation as it did not include all CVD conditions. Therefore, potentially the role of CVD was not fully accounted for in our results. The study size was also a limitation where it is likely to have affected the power of the study. Although small, this is however the first study of this nature, where we have assessed a vast panel of proteins in relation to D_LCO_ – an area where studies are lacking.

Lastly, the magnitude of associations between D_LCO_ and plasma proteins in this study do not imply that these proteins have use as clinical biomarkers. However, we did find larger differences in the levels of some proteins like MMP-12, GDF-15, TRAIL-R2 and U-PAR in those with D_LCO_< LLN vs. > LLN. Therefore, the mechanisms of the proteins associated with D_LCO_ give us an understanding of the potential pathophysiological mechanism that could link D_LCO_ to CVD outcomes.

We have found several CVD-linked plasma proteins to be significantly associated with D_LCO_, even after excluding subjects with obstruction on spirometry and some of these proteins were also linked to D_LCO_/V_A_%pred after excluding both subjects with obstruction on spirometry and abnormal alveolar volume. Therefore, likely explanations for these findings may be the pro-fibrotic and pro-inflammatory nature of many of the proteins causing changes in gas exchange.

## Supplementary Information

Supplementary Material 1

Supplementary Material 2

Supplementary Material 3

Supplementary Material 4

## Data Availability

The data that support the findings of this study are available from the corresponding author upon reasonable request.

## Of plasma proteins

FGF: 23-Fibroblast growth factor 23
MMP-12: Matrix metalloproteinase-12
GDF-15: Growth/differentiation factor 15
CCL20: C-C motif chemokine 20
IL: -6: Interleukin-6
FABP4: Fatty acid-binding protein, adipocyte
IL: 1ra: Interleukin-1 receptor antagonist
TRAIL: -R2: TNF-related apoptosis-inducing ligand receptor 2
U: PAR: -Urokinase-type plasminogen activator receptor
MMP1: -1: Matrix metalloproteinase-1
AM: Adrenomedullin
CSTB: Cystatin-B
CCL3: C-C motif chemokine 3
RAGE: Receptor for advanced glycation end products
REN: Renin
LEP: Leptin
SCF: Stem cell factor
SPON1: Spondin-1

## References

[1] Higbee DH, Granell R, Sanderson E, Davey Smith G, Dodd JW. Lung function & cardiovascular disease. A two sample Mendelian randomisation study. The European respiratory journal; 2021. 10.1183/13993003.03196-2020 33574079

[2] Rydell A, Nerpin E, Zhou X, Lind L, Lindberg E, Theorell Haglöw J, et al. Cardiovascular disease-linked plasma proteins are mainly associated with lung volume. ERJ Open Research 2023 9(2): 00321-2022. 10.1183/23120541.00321-2022. 37009020 10.1183/23120541.00321-2022 PMC10052712

[3] McNeill JN, Lee DH, Hwang SJ, Courchesne P, Yao C, Huan T, et al. Association of 71 cardiovascular disease-related plasma proteins with pulmonary function in the community. PLoS One. 2022;17(4):e0266523. 35390066 10.1371/journal.pone.0266523 PMC8989231

[4] Rydell A, Nowak C, Janson C, Lisspers K, Ställberg B, Iggman D, et al. Plasma proteomics and lung function in four community-based cohorts. Respir Med. 2021;176:106282. 33310204 10.1016/j.rmed.2020.106282

[5] Casselbrant A, Fedorowski A, Frantz S, Engström G, Wollmer P, Hamrefors V. Common physiologic and proteomic biomarkers in pulmonary and coronary artery disease. PLoS One. 2022;17(3):e0264376. 35263363 10.1371/journal.pone.0264376 PMC8906634

[6] Mehra R, Strohl KP. Chapter 14 - Evaluation and monitoring of respiratory function. In: Chokroverty S, editor. Sleep disorders medicine (Third Edition). Philadelphia: W.B. Saunders; 2009. pp. 188–97.

[7] Divinagracia JRC, Dummer J, Hancox RJ. Lung function and cardiovascular risk at age 45 in a cohort of the general population. Respir Med. 2024;222:107507. 38145722 10.1016/j.rmed.2023.107507

[8] Arcari A, Magnacca S, Bracone F, Costanzo S, Persichillo M, Di Castelnuovo A, et al. Relation between pulmonary function and 10-year risk for cardiovascular disease among healthy men and women in italy: the Moli-sani project. Eur J Prev Cardiol. 2013;20(5):862–71. 22609891 10.1177/2047487312447904

[9] Puri S, Baker BL, Dutka DP, Oakley CM, Hughes JMB, Cleland JGF. Reduced alveolar–capillary membrane diffusing capacity in chronic heart failure. Circulation. 1995;91(11):2769–74. 7758183 10.1161/01.cir.91.11.2769

[10] Frantz S, Nihlen U, Dencker M, Engstrom G, Lofdahl CG, Wollmer P. Atherosclerotic plaques in the internal carotid artery and associations with lung function assessed by different methods. Clin Physiol Funct Imaging. 2012;32(2):120–5. 22296632 10.1111/j.1475-097X.2011.01065.x

[11] Lee JS, Rosengart MR, Kondragunta V, Zhang Y, McMurray J, Branch RA, et al. Inverse association of plasma IL-13 and inflammatory chemokines with lung function impairment in stable COPD: a cross-sectional cohort study. Respir Res. 2007;8(1):64. 17868461 10.1186/1465-9921-8-64 PMC2064925

[12] Rosas IO, Richards TJ, Konishi K, Zhang Y, Gibson K, Lokshin AE, et al. MMP1 and MMP7 as potential peripheral blood biomarkers in idiopathic pulmonary fibrosis. PLoS Med. 2008;5(4):e93. 18447576 10.1371/journal.pmed.0050093 PMC2346504

[13] Kraen M, Frantz S, Nihlén U, Engström G, Löfdahl CG, Wollmer P, et al. Fibroblast growth factor 23 is an independent marker of COPD and is associated with impairment of pulmonary function and diffusing capacity. Respir Med. 2021;182:106404. 33895626 10.1016/j.rmed.2021.106404

[14] Kraen M, Frantz S, Nihlén U, Engström G, Löfdahl CG, Wollmer P, et al. Matrix metalloproteinases in COPD and atherosclerosis with emphasis on the effects of smoking. PLoS One. 2019;14(2):e0211987. 30789935 10.1371/journal.pone.0211987 PMC6383934

[15] Montnemery P, Adelroth E, Heuman K, Johannisson A, Johansson S-Å, Lindholm L-H, et al. Prevalence of obstructive lung diseases and respiratory symptoms in southern Sweden. Respir Med. 1998;92(12):1337–45. 10197227 10.1016/s0954-6111(98)90139-1

[16] Nihlén U, Nyberg P, Montnémery P, Löfdahl C-G. Influence of family history and smoking habits on the incidence of self-reported physician’s diagnosis of COPD. Respir Med. 2004;98(3):263–70. 15002763 10.1016/j.rmed.2003.10.006

[17] Quanjer PH, Tammeling GJ, Cotes JE, Pedersen OF, Peslin R, Yernault JC. Lung volumes and forced ventilatory flows. Report working party standardization of lung function tests, European community for steel and coal. Official statement of the European respiratory society. Eur Respir J Suppl. 1993;16:5–40. 8499054

[18] Cotes JE, Chinn DJ, Quanjer PH, Roca J, Yernault J-C. Standardization of the measurement of transfer factor (diffusing capacity). Eur Respir J. 1993;6(Suppl 16):41–52. 24576916 10.1183/09041950.041s1693

[19] MacIntyre N, Crapo RO, Viegi G, Johnson DC, van der Grinten CPM, Brusasco V, et al. Standardisation of the single-breath determination of carbon monoxide uptake in the lung. Eur Respir J. 2005;26(4):720–35. 16204605 10.1183/09031936.05.00034905

[20] Stanojevic S, Graham BL, Cooper BG, Thompson Bruce R, Carter KW, Francis RW, et al. Official ERS technical standards: global lung function initiative reference values for the carbon monoxide transfer factor for Caucasians. Eur Respir J. 2017;50(3):1700010. 28893868 10.1183/13993003.00010-2017

[21] Assarsson E, Lundberg M, Holmquist G, Björkesten J, Thorsen SB, Ekman D, et al. Homogenous 96-plex PEA immunoassay exhibiting high sensitivity, specificity, and excellent scalability. PLoS One. 2014;9(4):e95192-e. 24755770 10.1371/journal.pone.0095192 PMC3995906

[22] Lundberg M, Eriksson A, Tran B, Assarsson E, Fredriksson S. Homogeneous antibody-based proximity extension assays provide sensitive and specific detection of low-abundant proteins in human blood. Nucleic Acids Res. 2011;39(15):e102–e. 21646338 10.1093/nar/gkr424 PMC3159481

[23] R Core Team. (2025) _R: A Language and Environment for Statistical Computing_. R Foundation for Statistical Computing, Vienna, Austria. [Available from: <https://www.R-project.org/

[24] Kraen M, Frantz S, Nihlén U, Engström G, Löfdahl C-G, Wollmer P, et al. Brain natriuretic peptide levels in middle aged subjects with normal left ventricular function in relation to mild–moderate COPD. Clin Respir J. 2018;12(3):1061–7. 28294547 10.1111/crj.12628

[25] Krick S, Grabner A, Baumlin N, Yanucil C, Helton S, Grosche A, et al. Fibroblast growth factor 23 and Klotho contribute to airway inflammation. Eur Respir J. 2018;52(1):1800236. 29748308 10.1183/13993003.00236-2018 PMC6044452

[26] Lappalainen U, Whitsett JA, Wert SE, Tichelaar JW, Bry K. Interleukin-1β causes pulmonary inflammation, emphysema, and airway remodeling in the adult murine lung. Am J Respir Cell Mol Biol. 2005;32(4):311–8. 15668323 10.1165/rcmb.2004-0309OC

[27] Marthi A, Donovan K, Haynes R, Wheeler DC, Baigent C, Rooney CM, et al. Fibroblast growth factor-23 and risks of cardiovascular and noncardiovascular diseases: a meta-analysis. J Am Soc Nephrol. 2018;29(7):2015–27. 29764921 10.1681/ASN.2017121334 PMC6050929

[28] Donovan K, Herrington WG, Paré G, Pigeyre M, Haynes R, Sardell R, et al. Fibroblast growth factor-23 and risk of cardiovascular diseases: a mendelian randomization study. Clin J Am Soc Nephrol. 2023;18(1):17–27. 36719157 10.2215/CJN.05080422 PMC7614195

[29] McGarry Houghton A. Matrix metalloproteinases in destructive lung disease. Matrix Biol. 2015;44– 46:167–74. 10.1016/j.matbio.2015.02.002 25686691

[30] Traylor M, Mäkelä KM, Kilarski LL, Holliday EG, Devan WJ, Nalls MA, et al. A novel MMP12 locus is associated with large artery atherosclerotic stroke using a genome-wide age-at-onset informed approach. PLoS Genet. 2014;10(7):e1004469. 25078452 10.1371/journal.pgen.1004469 PMC4117446

[31] Goncalves I, Bengtsson E, Colhoun HM, Shore AC, Palombo C, Natali A, et al. Elevated plasma levels of MMP-12 are associated with atherosclerotic burden and symptomatic cardiovascular disease in subjects with type 2 diabetes. Arterioscler Thromb Vasc Biol. 2015;35(7):1723–31. 25953645 10.1161/ATVBAHA.115.305631

[32] Buckley LF, Abbate A. Interleukin-1 blockade in cardiovascular diseases: a clinical update. Eur Heart J. 2018;39(22):2063–9. 29584915 10.1093/eurheartj/ehy128

[33] Mao XQ, Kawai M, Yamashita T, Enomoto T, Dake Y, Sasaki S, et al. Imbalance production between interleukin-1beta (IL-1beta) and IL-1 receptor antagonist (IL-1Ra) in bronchial asthma. Biochem Biophys Res Commun. 2000;276(2):607–12. 11027520 10.1006/bbrc.2000.3516

[34] Sapey E, Ahmad A, Bayley D, Newbold P, Snell N, Rugman P, et al. Imbalances between interleukin-1 and tumor necrosis factor agonists and antagonists in stable COPD. J Clin Immunol. 2009;29(4):508–16. 19291375 10.1007/s10875-009-9286-8

[35] Su JH, Luo MY, Liang N, Gong SX, Chen W, Huang WQ, et al. Interleukin-6: a novel target for cardio-cerebrovascular diseases. Front Pharmacol. 2021;12:745061. 34504432 10.3389/fphar.2021.745061 PMC8421530

[36] Gimeno D, Delclos GL, Ferrie JE, De Vogli R, Elovainio M, Marmot MG, et al. Association of CRP and IL-6 with lung function in a middle-aged population initially free from self-reported respiratory problems: the Whitehall II study. Eur J Epidemiol. 2011;26(2):135–44. 21293970 10.1007/s10654-010-9526-5 PMC3199309

[37] Lee JH, Jang JH, Park JH, Jang HJ, Park CS, Lee S, et al. The role of interleukin-6 as a prognostic biomarker for predicting acute exacerbation in interstitial lung diseases. PLoS One. 2021;16(7):e0255365. 34314462 10.1371/journal.pone.0255365 PMC8315549

[38] Husebø GR, Grønseth R, Lerner L, Gyuris J, Hardie JA, Bakke PS, et al. Growth differentiation factor-15 is a predictor of important disease outcomes in patients with COPD. Eur Respir J. 2017-1601298. 10.1183/13993003.01298-2016. 28298399 10.1183/13993003.01298-2016

[39] Wollert KC, Kempf T, Wallentin L. Growth differentiation factor 15 as a biomarker in cardiovascular disease. Clin Chem. 2017;63(1):140–51. 28062617 10.1373/clinchem.2016.255174

[40] Wan Y, Fu J. GDF15 as a key disease target and biomarker: linking chronic lung diseases and ageing. Mol Cell Biochem. 2024;479(3):453–66. 37093513 10.1007/s11010-023-04743-x PMC10123484

[41] Zhang Y, Jiang M, Nouraie M, Roth MG, Tabib T, Winters S, et al. GDF15 is an epithelial-derived biomarker of idiopathic pulmonary fibrosis. Am J Physiol Lung Cell Mol Physiol. 2019;317(4):L510-l21. 31432710 10.1152/ajplung.00062.2019 PMC6842909

[42] Bao X, Borné Y, Yin S, Niu K, Orho-Melander M, Nilsson J, et al. The associations of self-rated health with cardiovascular risk proteins: a proteomics approach. Clin Proteomics. 2019;16(1):40. 31832026 10.1186/s12014-019-9258-9 PMC6859604

[43] Demedts IK, Bracke KR, Pottelberge GV, Testelmans D, Verleden GM, Vermassen FE, et al. Accumulation of dendritic cells and increased CCL20 levels in the airways of patients with chronic obstructive pulmonary disease. Am J Respir Crit Care Med. 2007;175(10):998–1005. 17332482 10.1164/rccm.200608-1113OC

[44] Sun D, Ouyang Y, Gu Y, Liu X. Cigarette smoke-induced chronic obstructive pulmonary disease is attenuated by CCL20-blocker: a rat model. Croat Med J. 2016;57(4):363–70. 27586551 10.3325/cmj.2016.57.363 PMC5048234

[45] Shore SA. Obesity and asthma: cause for concern. Curr Opin Pharmacol. 2006;6(3):230–6. 16530012 10.1016/j.coph.2006.01.004

[46] Park HJ, Park SE, Park CY, Lim SY, Lee WY, Oh KW, et al. The relationship between serum fatty-acid binding protein 4 level and lung function in Korean subjects with normal ventilatory function. BMC Pulm Med. 2016;16:34. 26887419 10.1186/s12890-016-0190-8 PMC4757985

[47] Aslani MR, Ghazaei Z, Ghobadi H. Correlation of serum fatty acid binding protein-4 and interleukin-6 with airflow limitation and quality of life in stable and acute exacerbation of COPD. Turk J Med Sci. 2020;50(2):337–45. 31905499 10.3906/sag-1909-9 PMC7164746

[48] Makowski L, Brittingham KC, Reynolds JM, Suttles J, Hotamisligil GS. The fatty acid-binding protein, aP2, coordinates macrophage cholesterol trafficking and inflammatory activity. Macrophage expression of aP2 impacts peroxisome proliferator-activated receptor gamma and IkappaB kinase activities. J Biol Chem. 2005;280(13):12888–95. 15684432 10.1074/jbc.M413788200 PMC3493120

[49] Ghelfi E, Yu CW, Elmasri H, Terwelp M, Lee CG, Bhandari V, et al. Fatty acid binding protein 4 regulates VEGF-induced airway angiogenesis and inflammation in a Transgenic mouse model: implications for asthma. Am J Pathol. 2013;182(4):1425–33. 23391391 10.1016/j.ajpath.2012.12.009 PMC3620401

[50] Egervall K, Rosso A, Elmståhl S. Association between cardiovascular disease- and inflammation-related serum biomarkers and poor lung function in elderly. Clin Proteomics. 2021;18(1):23. 34583636 10.1186/s12014-021-09329-7 PMC8480099

[51] Can Ü, Güzelant A, Yerlikaya FH, Yosunkaya Ş. The role of serum soluble Urokinase-Type plasminogen activator receptor in stable chronic obstructive pulmonary disease. J Investig Med. 2014;62(7):938–43. 25127435 10.1097/JIM.0000000000000105

[52] Grisanti LA. TRAIL and its receptors in cardiac diseases. Front Physiol. 2023;14:1256852. 37621762 10.3389/fphys.2023.1256852 PMC10445540

[53] Wu Y, Shen Y, Zhang J, Wan C, Wang T, Xu D, et al. Increased serum TRAIL and DR5 levels correlated with lung function and inflammation in stable COPD patients. Int J Chron Obstruct Pulmon Dis. 2015;10:2405–12. 26609227 10.2147/COPD.S92260 PMC4644161

[54] Zaigham S, Dencker M, Karlsson MK, Thorsson O, Wollmer P. Lung function is associated with tumour necrosis factor-related apoptosis-inducing ligand (TRAIL) levels in school-aged children. Respir Med. 2021;176:106235. 33249302 10.1016/j.rmed.2020.106235

[55] Cohn JN. Role of the renin-angiotensin system in cardiovascular disease. Cardiovasc Drugs Ther. 2010;24(4):341–4. 20454841 10.1007/s10557-010-6230-3

[56] Gupta D, Kumar A, Mandloi A, Shenoy V. Renin angiotensin aldosterone system in pulmonary fibrosis: pathogenesis to therapeutic possibilities. Pharmacol Res. 2021;174:105924. 34607005 10.1016/j.phrs.2021.105924

[57] Shah AM, Myhre PL, Arthur V, Dorbala P, Rasheed H, Buckley LF, et al. Large scale plasma proteomics identifies novel proteins and protein networks associated with heart failure development. Nat Commun. 2024;15(1):528. 38225249 10.1038/s41467-023-44680-3 PMC10789789

