# Supplementary figures and images for: Diffusing capacity for carbon monoxide is significantly associated with cardiovascular disease-related plasma proteins, independently of obstruction
Source: Clin Proteomics. 2026 Feb 1;23:11. doi: 10.1186/s12014-026-09584-6 (PMC12927244; doi:10.1186/s12014-026-09584-6)

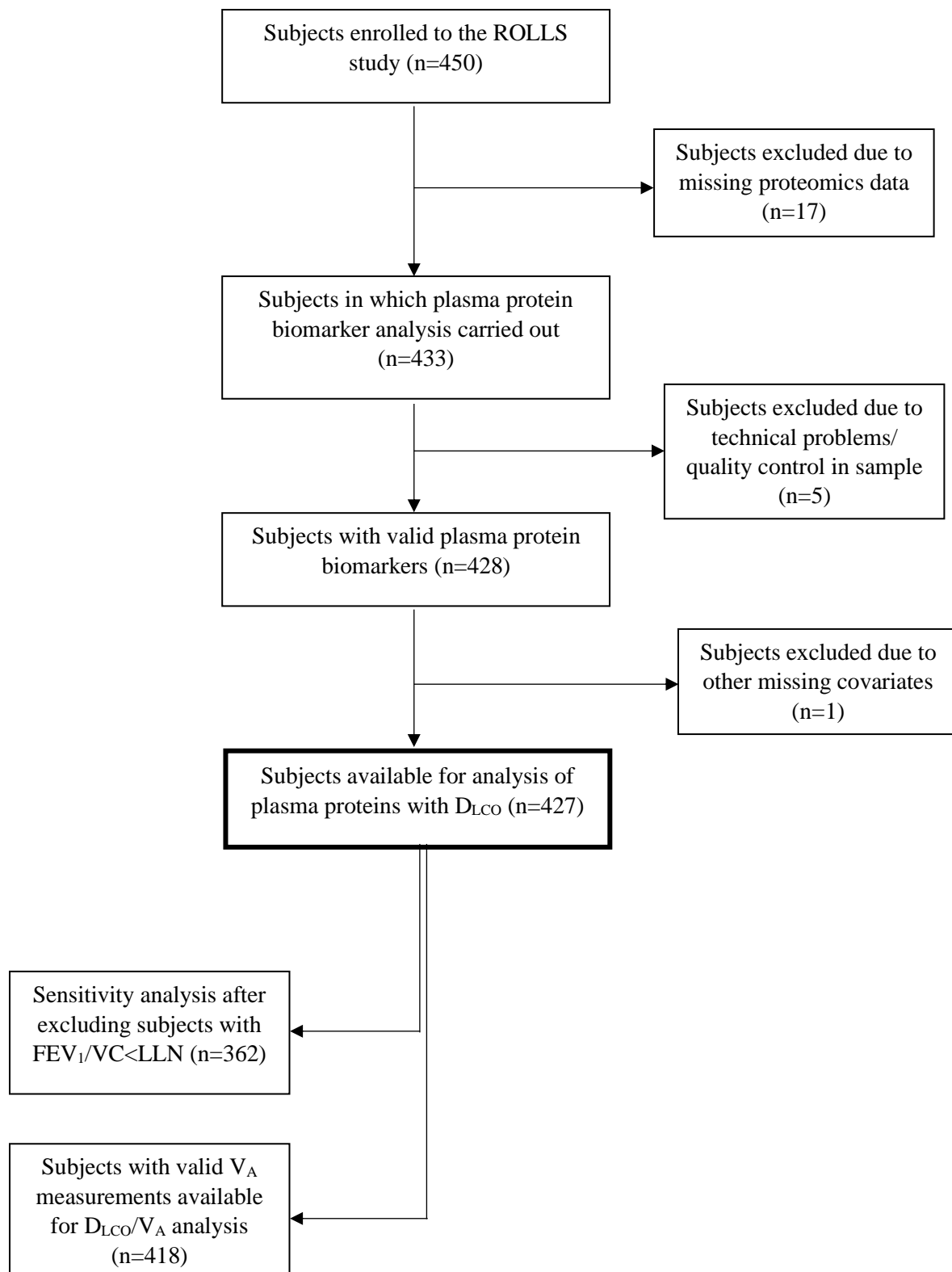

Supplement: Supplementary file 1 — Supplementary Material 1 [file 12014_2026_9584_MOESM1_ESM.pdf]

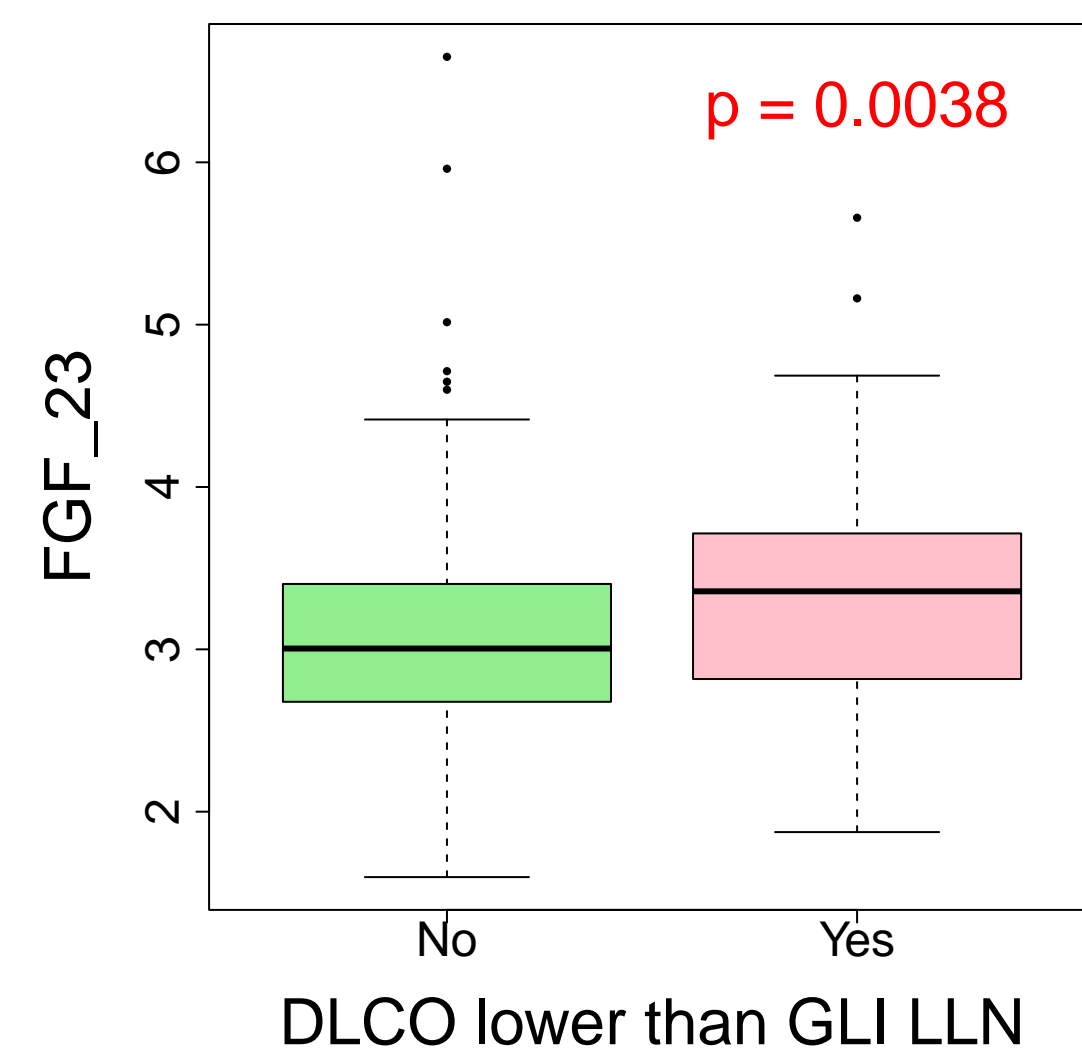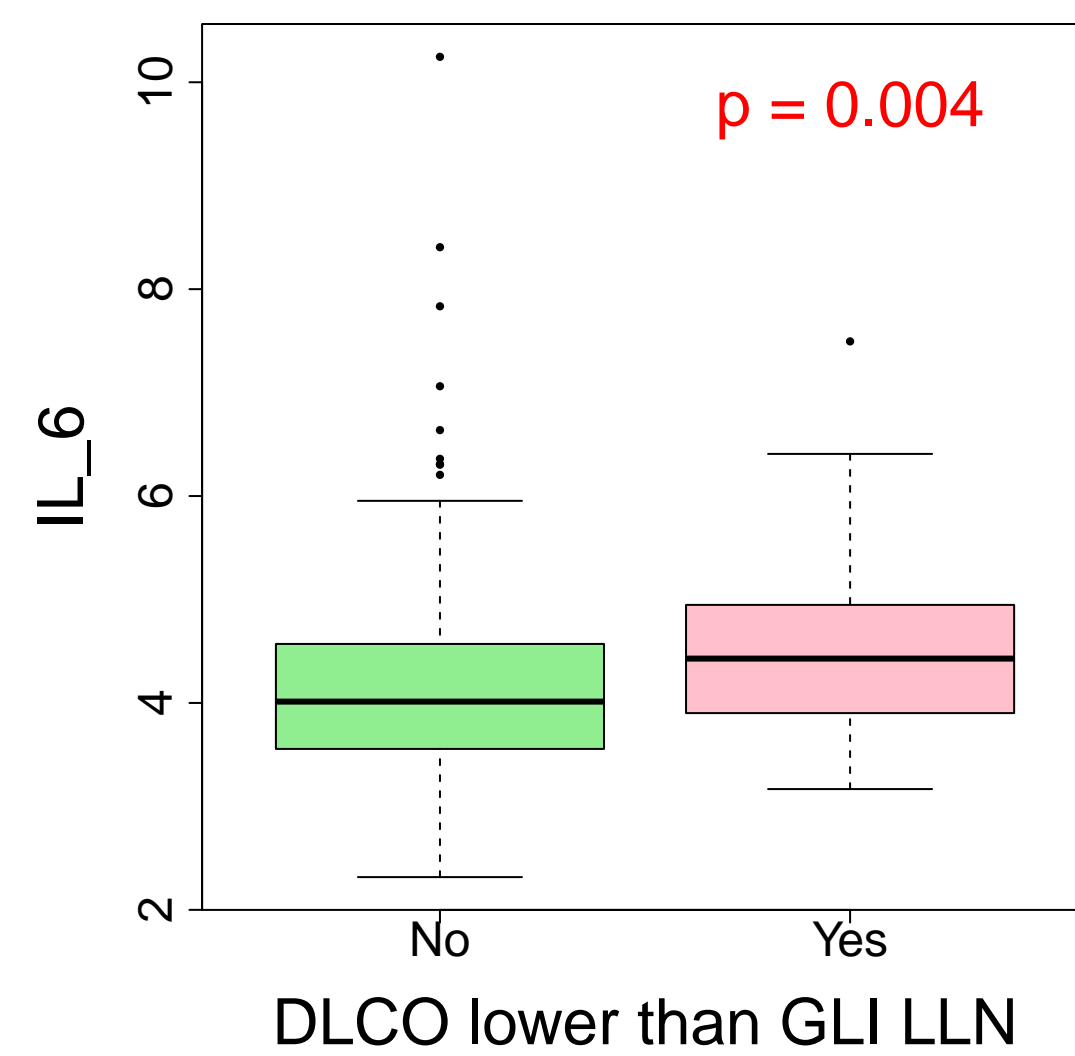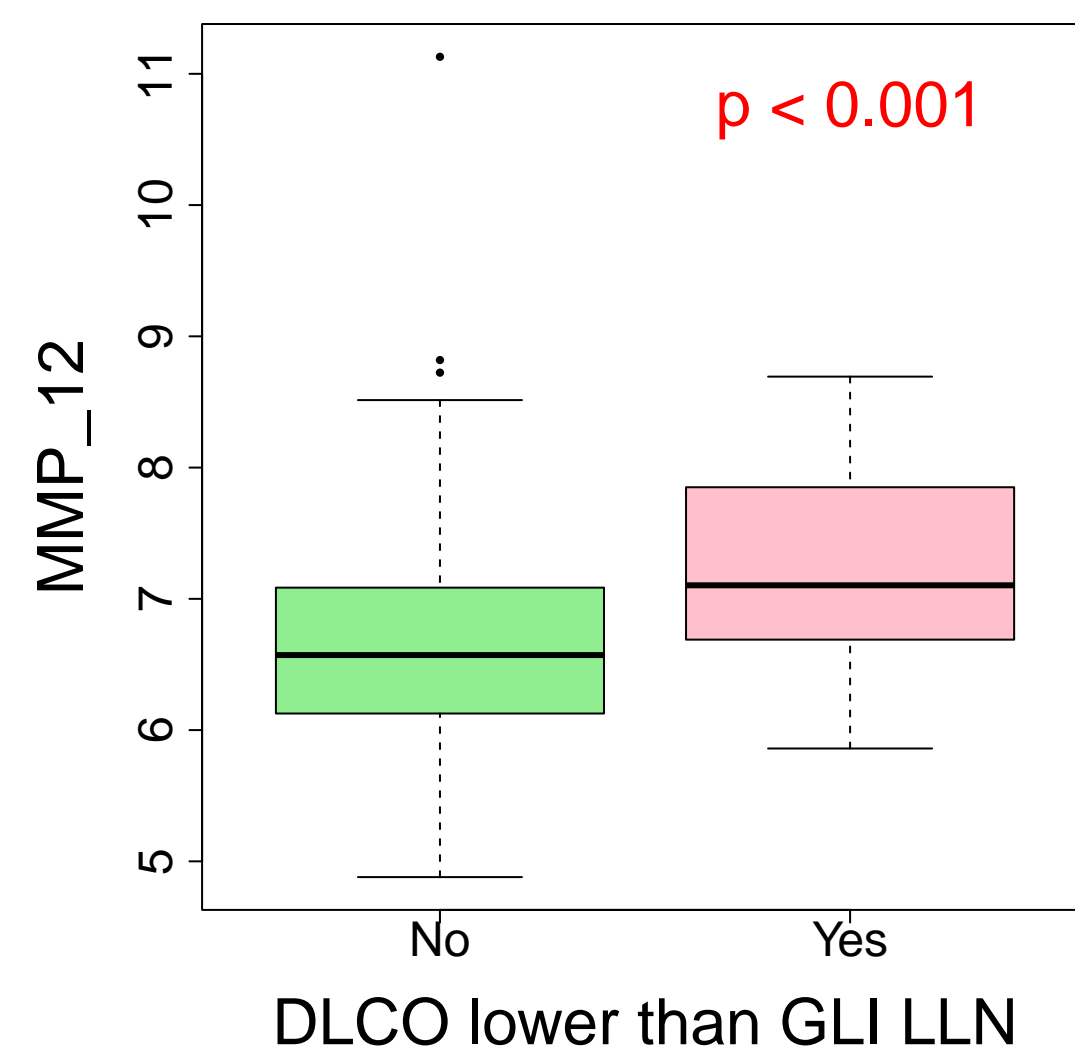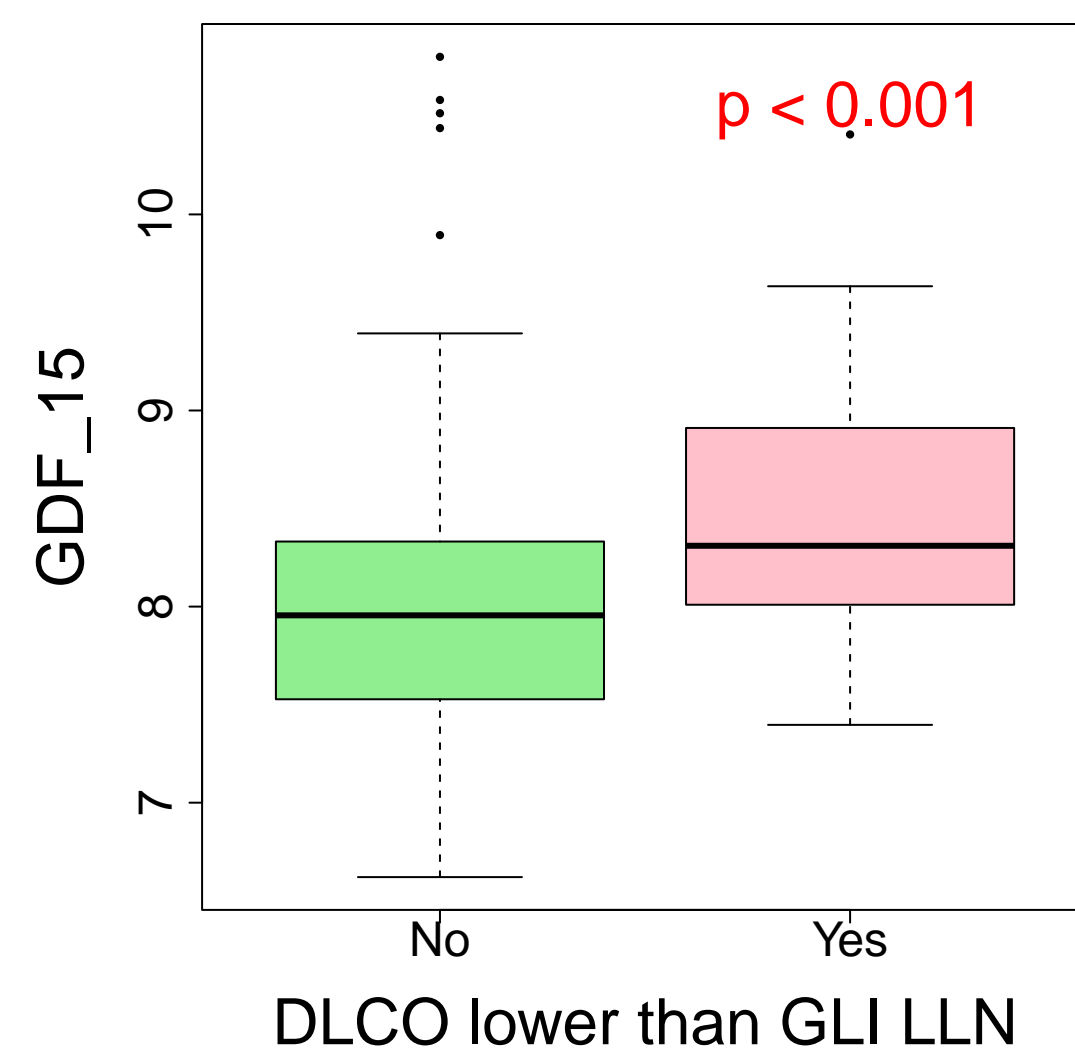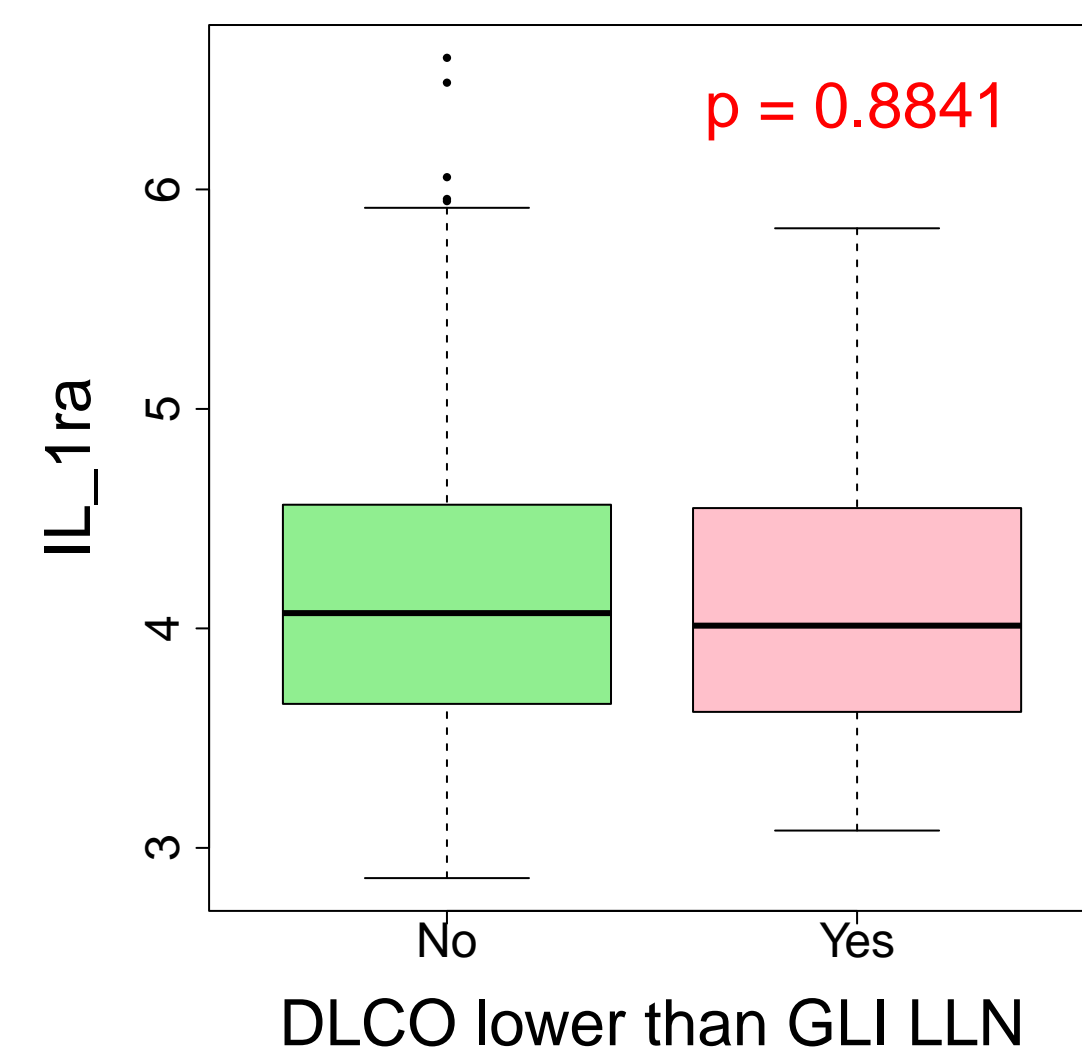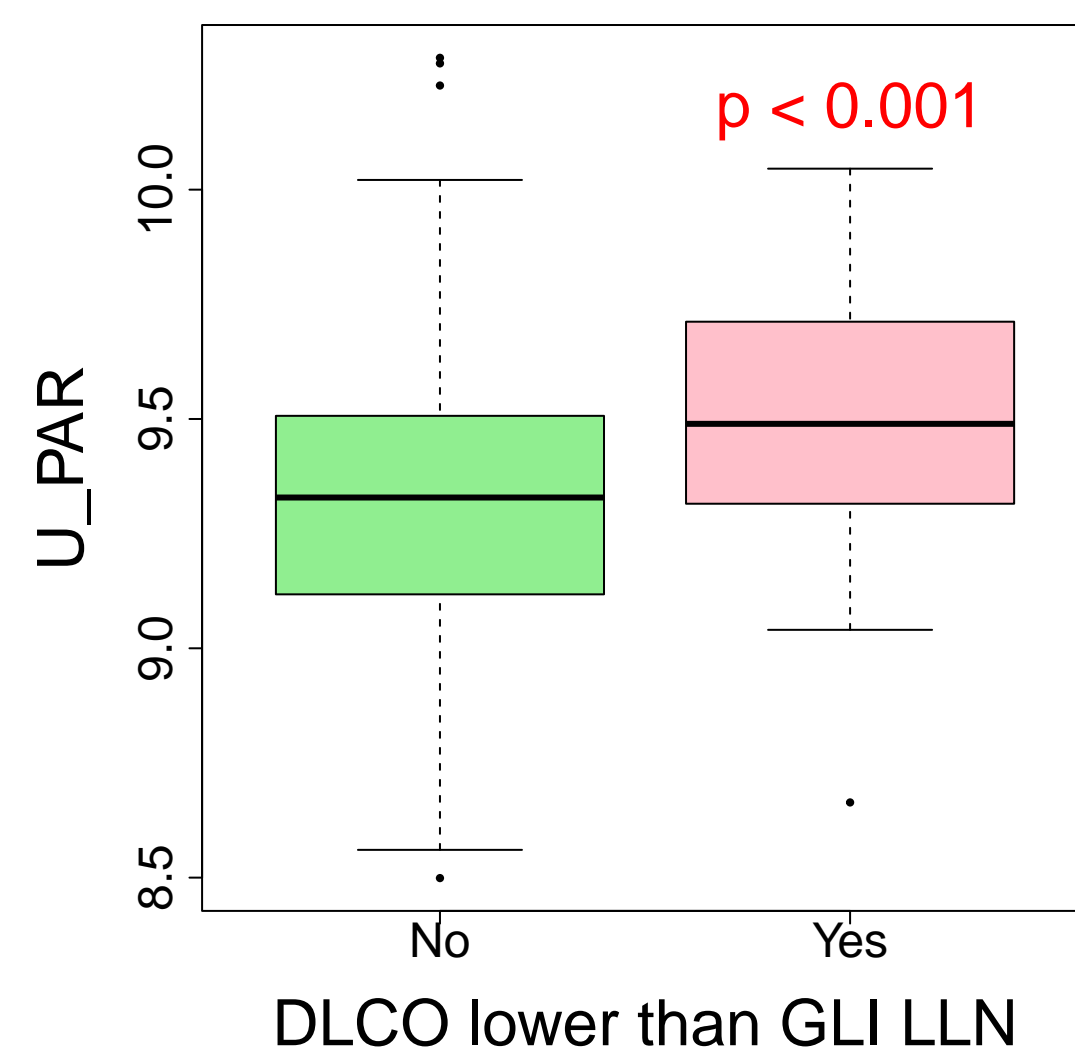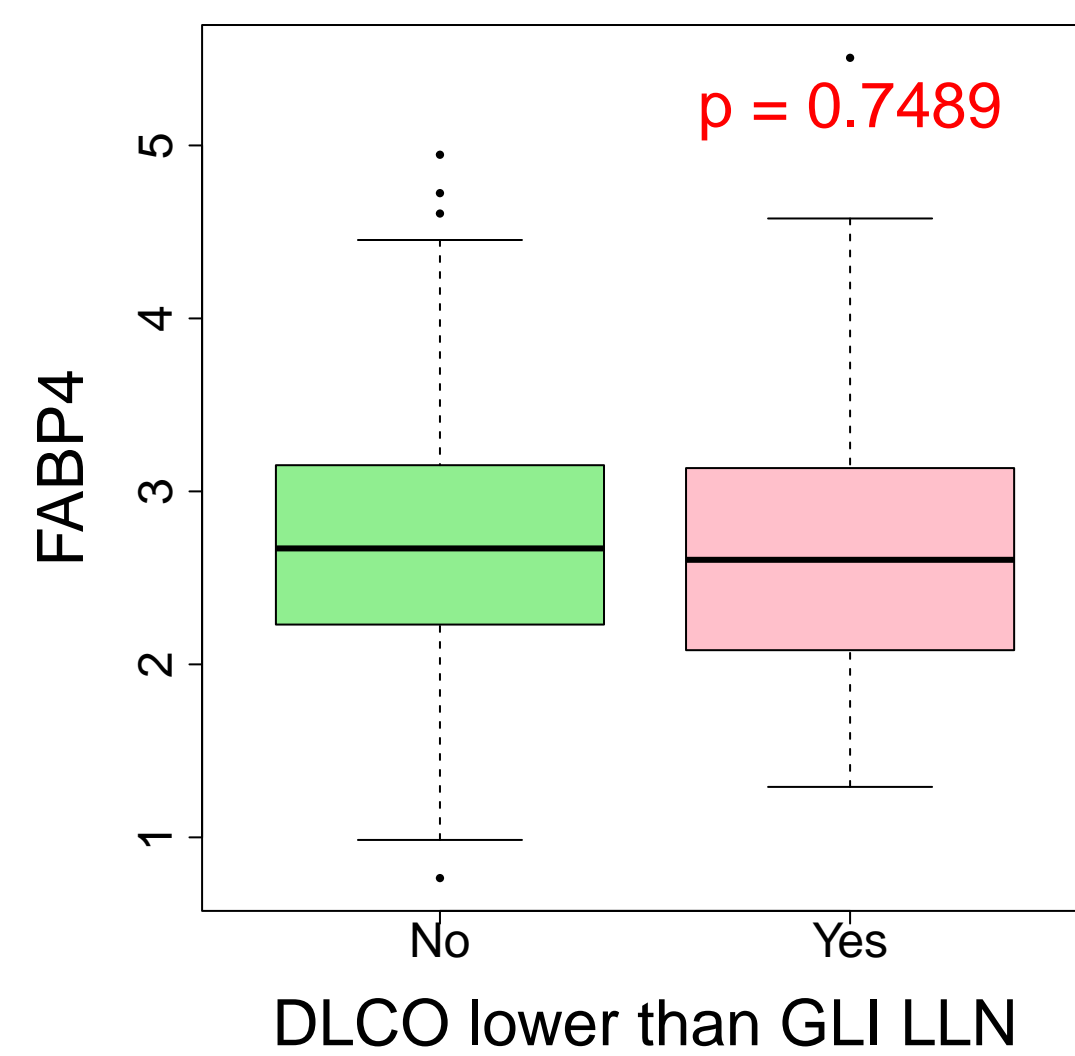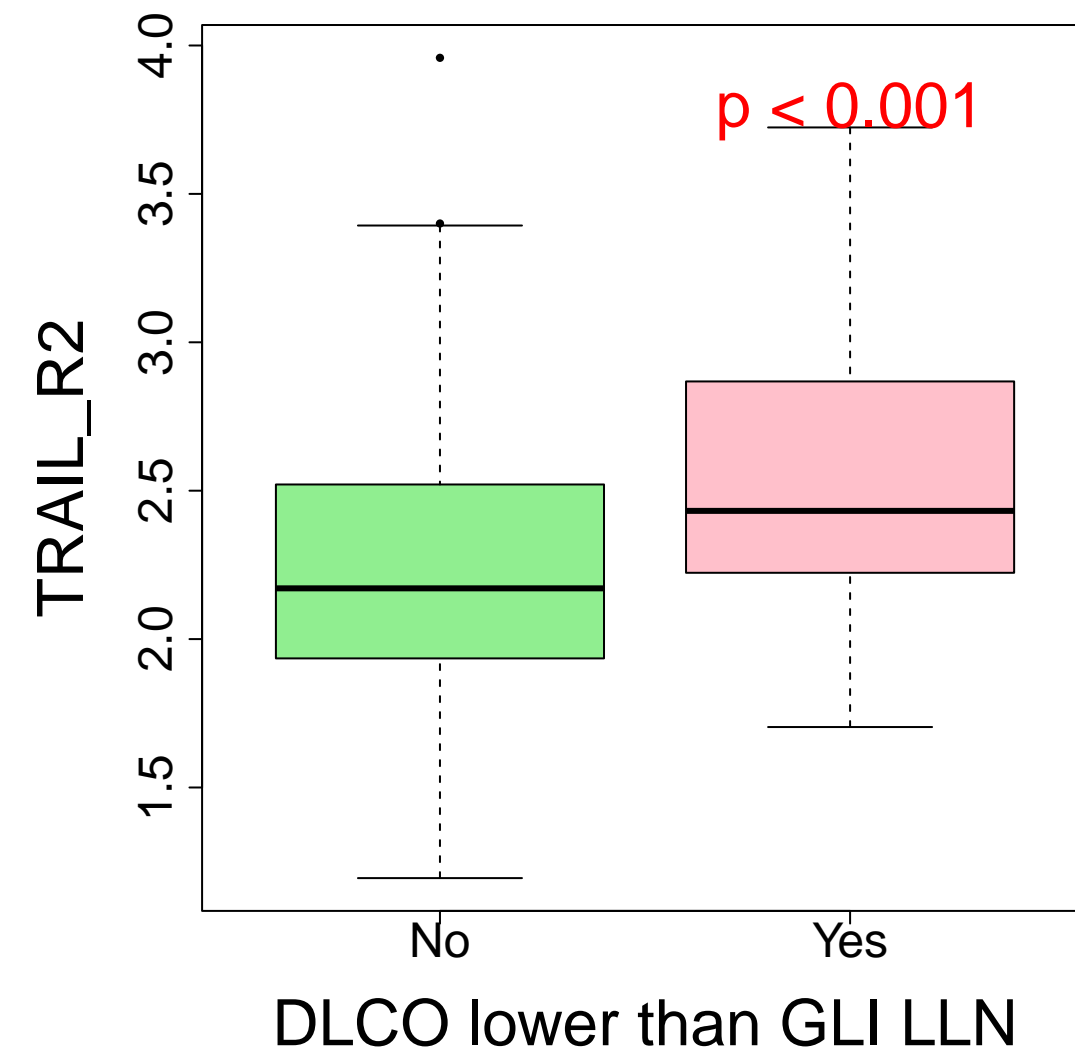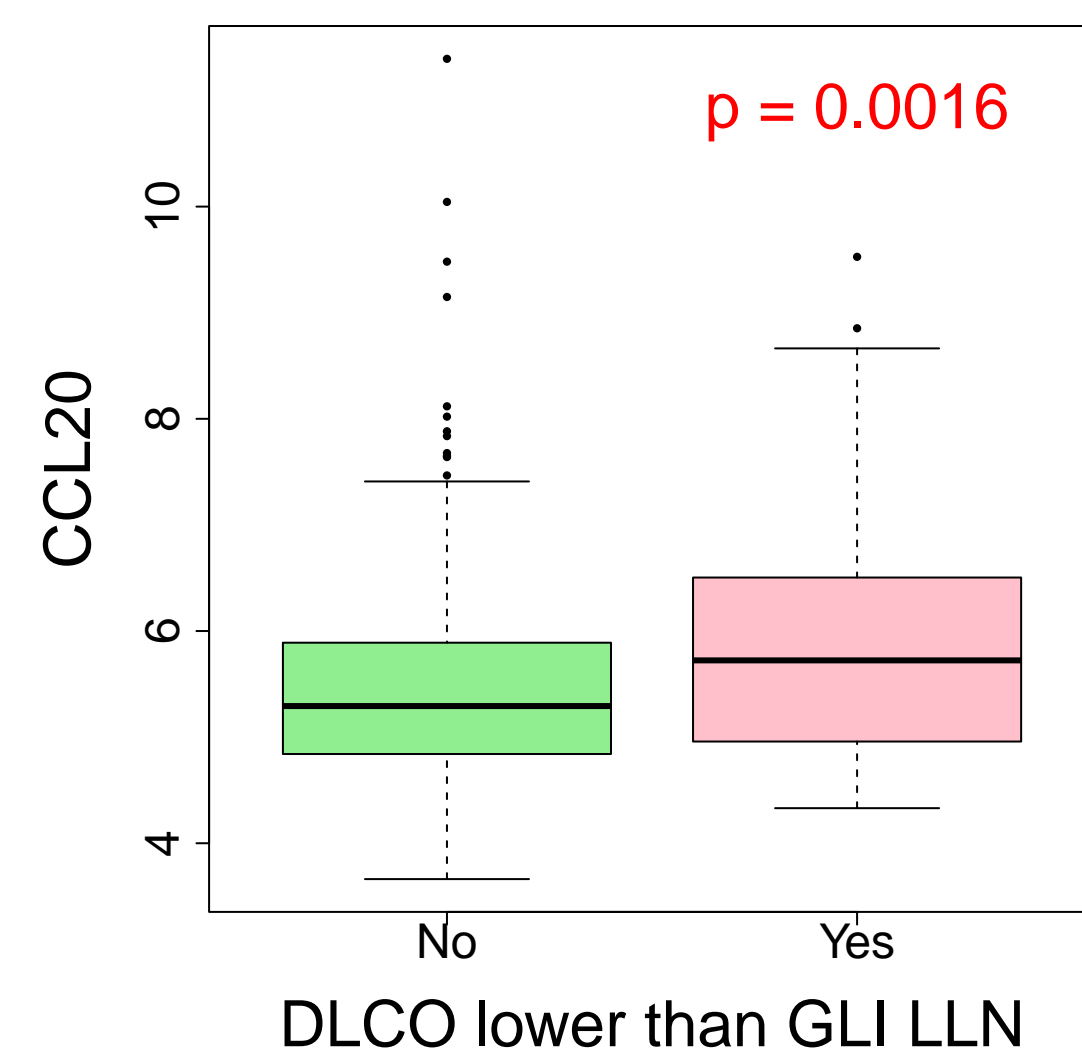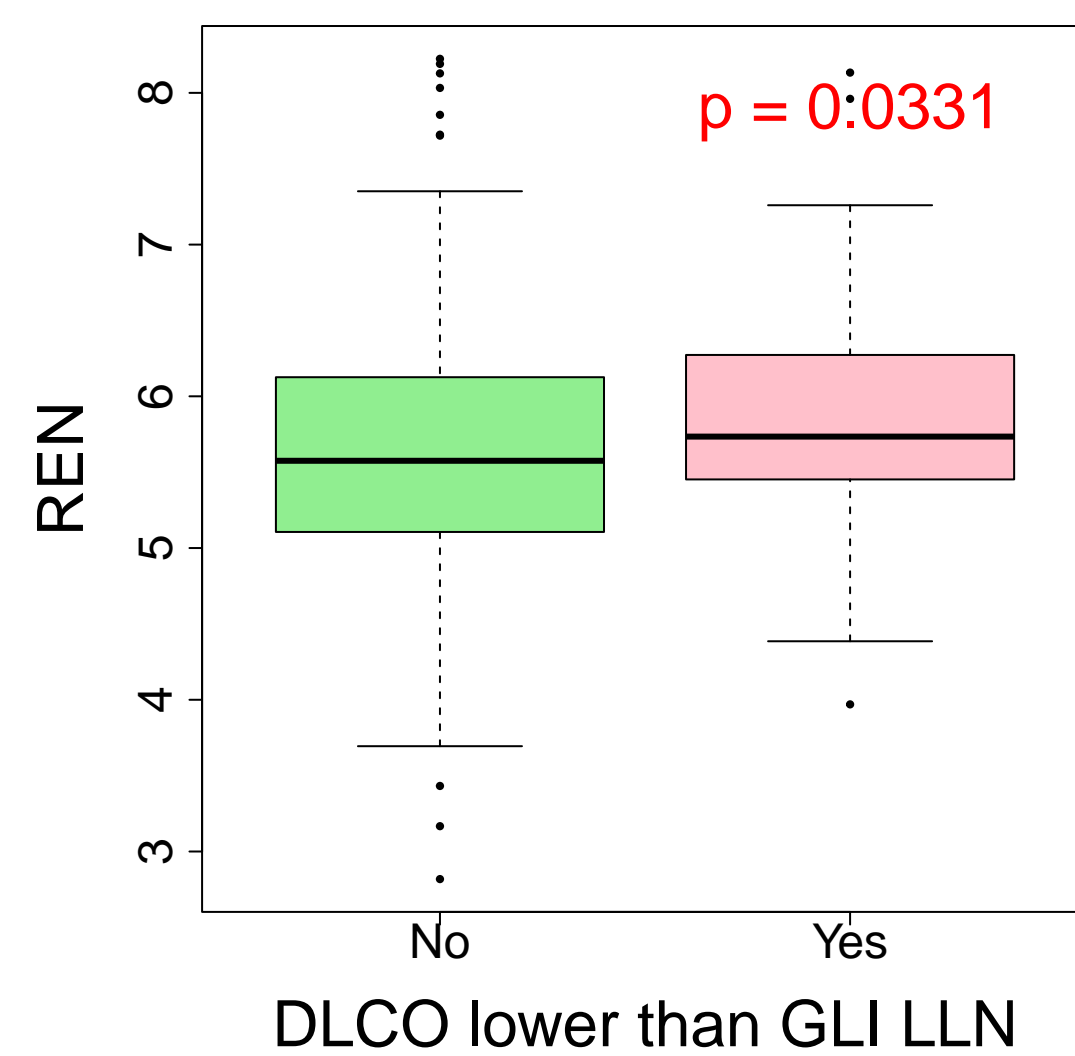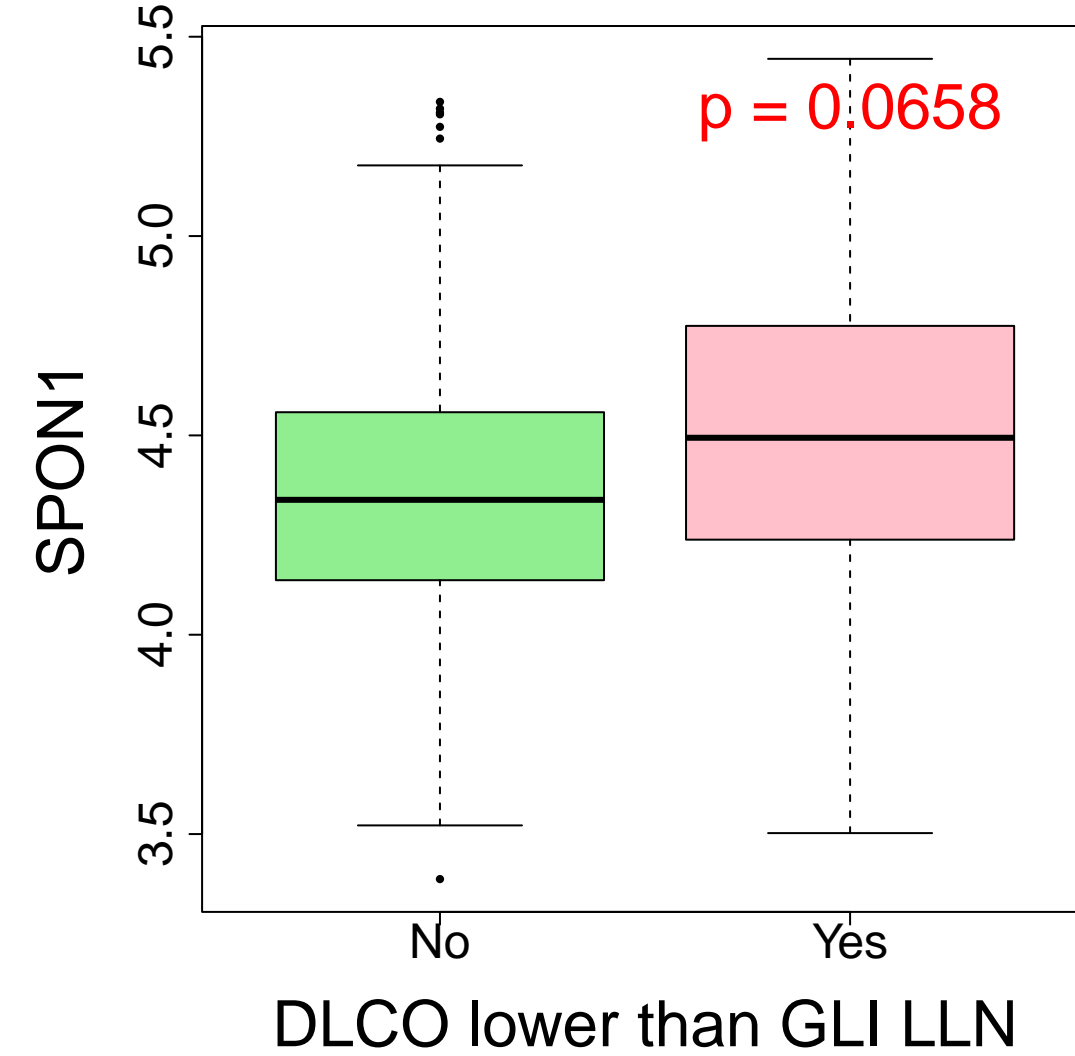

Supplement: Supplementary file 2 — Supplementary Material 2 [file 12014_2026_9584_MOESM2_ESM.pdf]

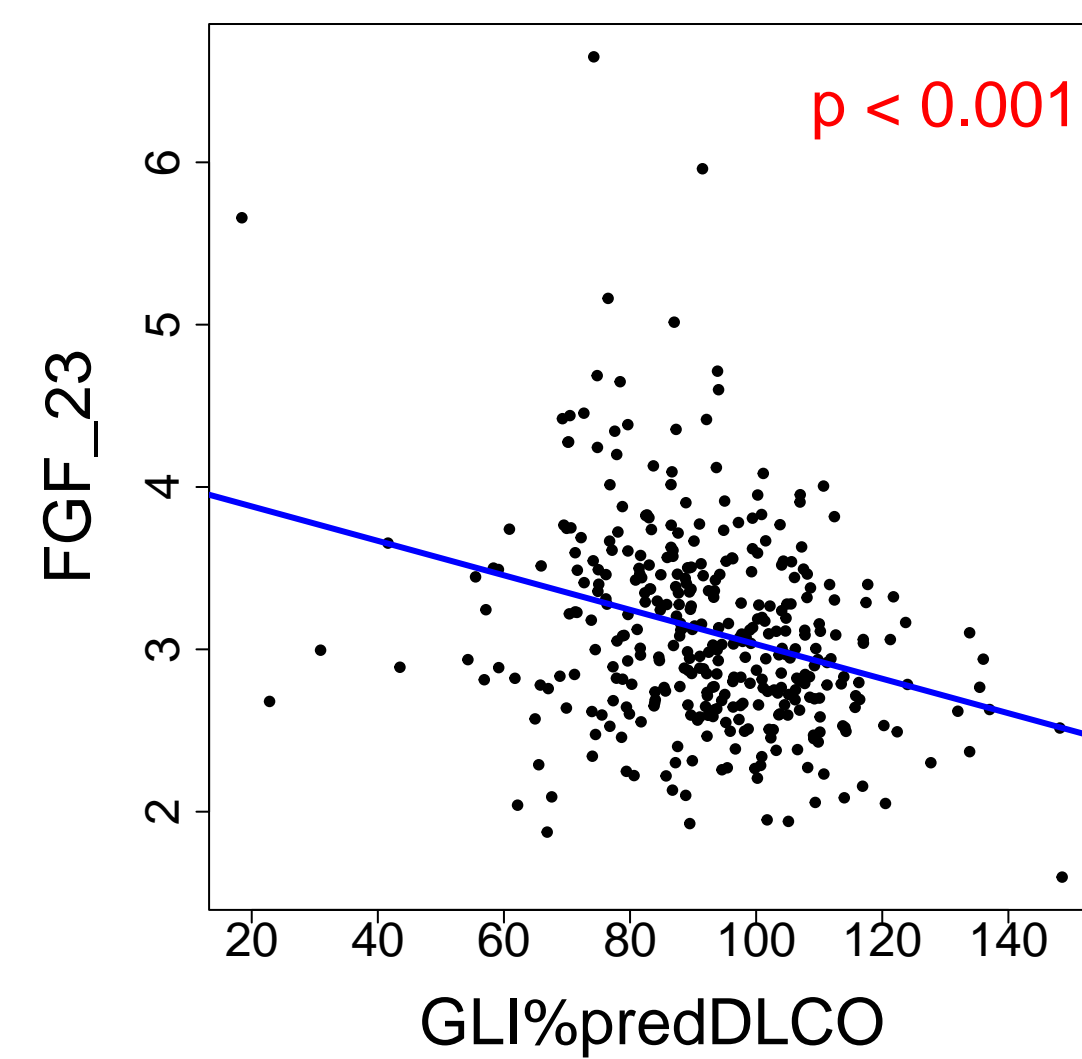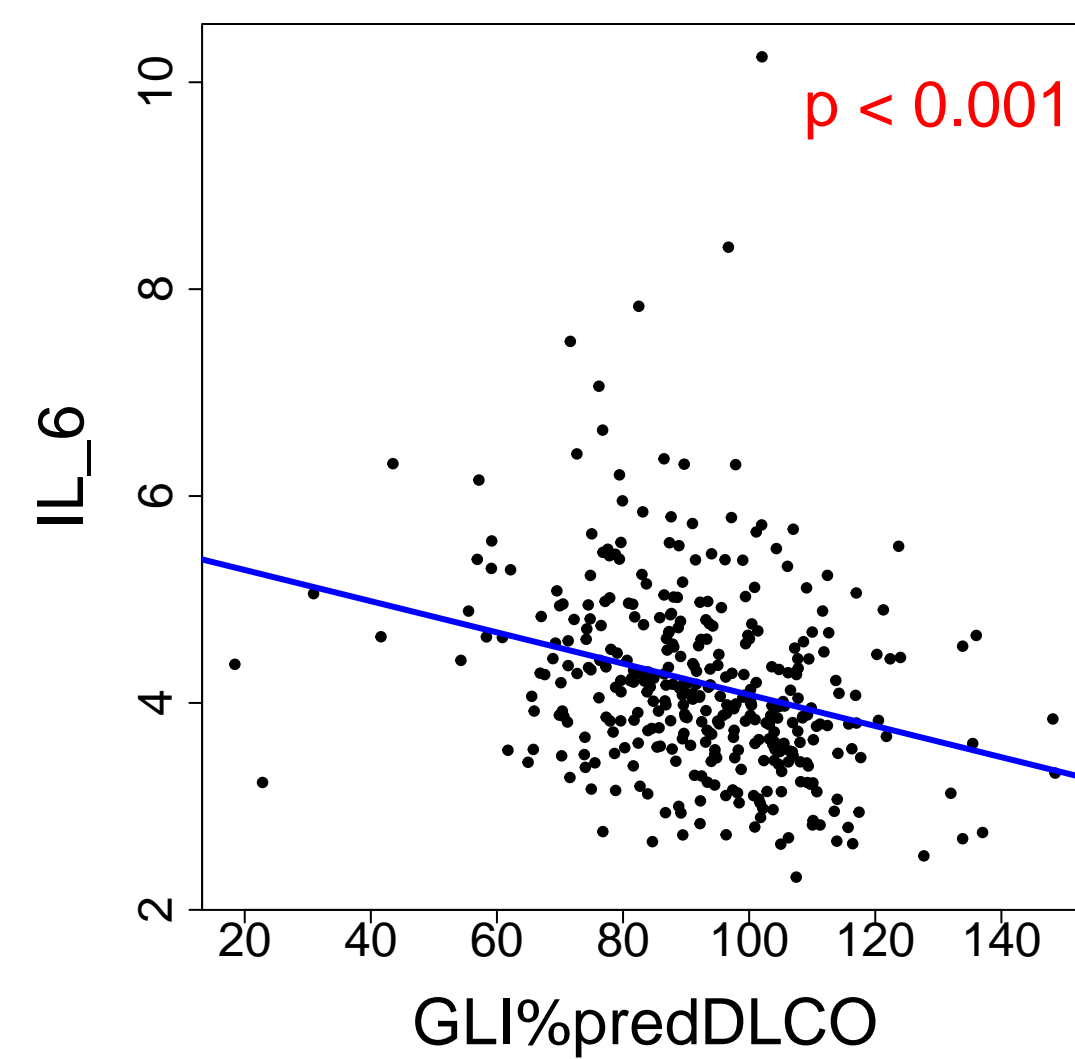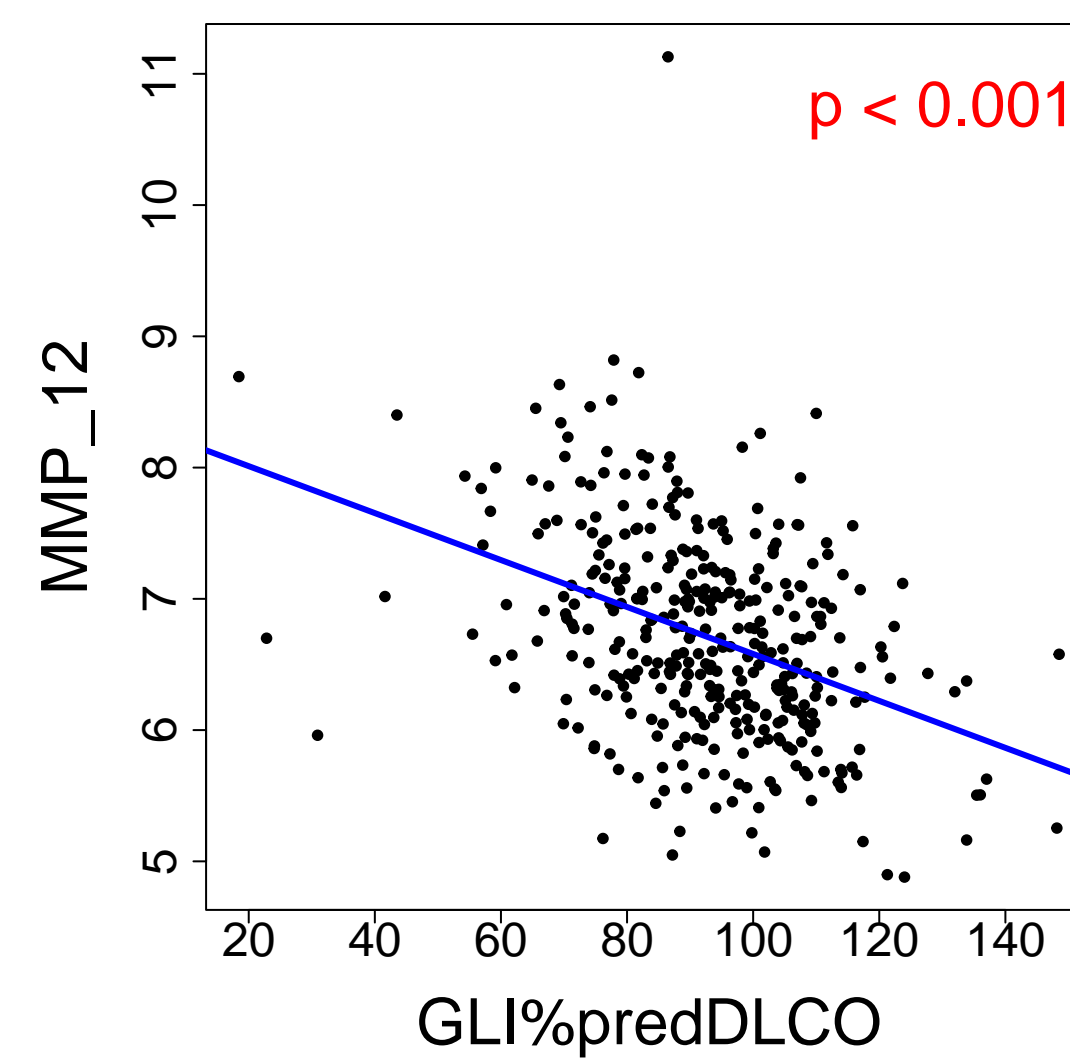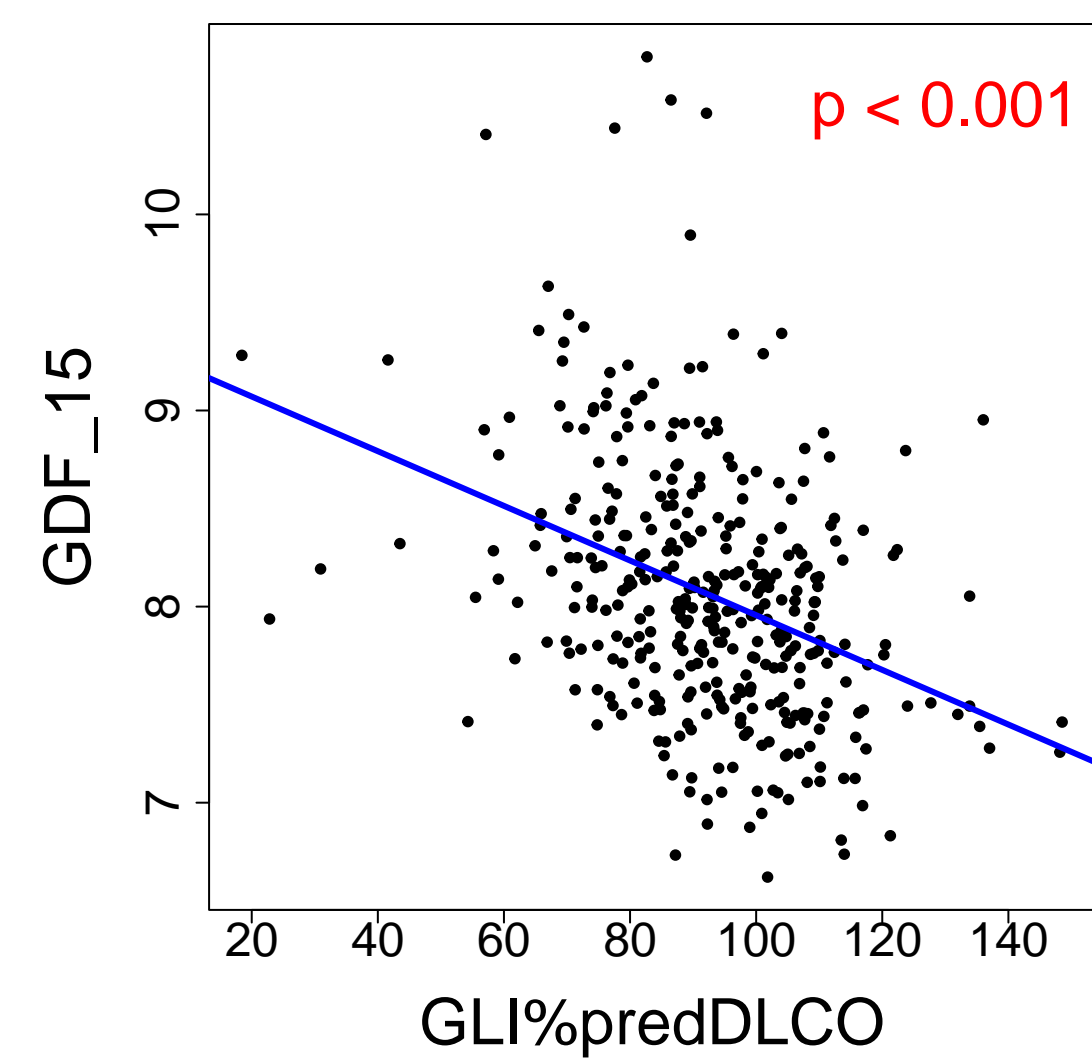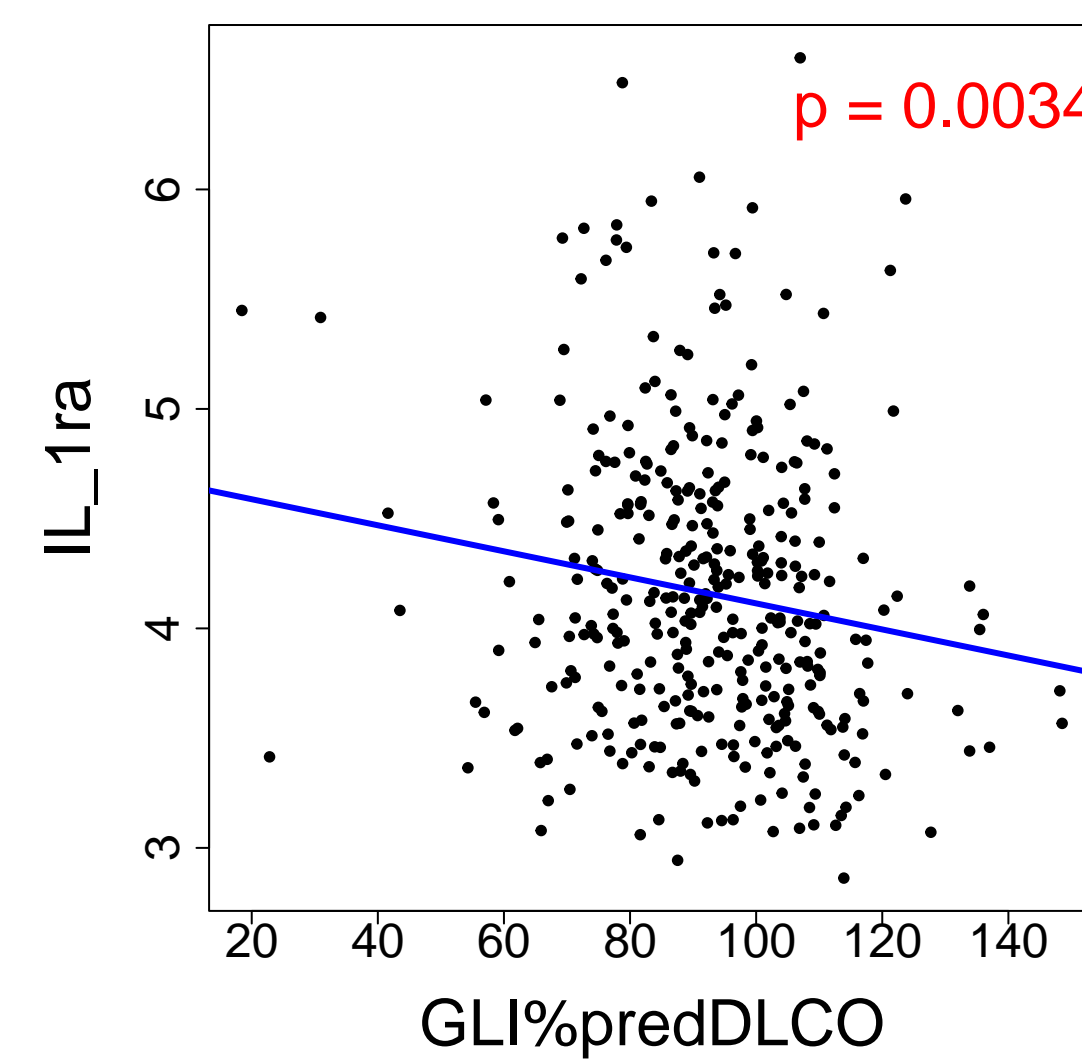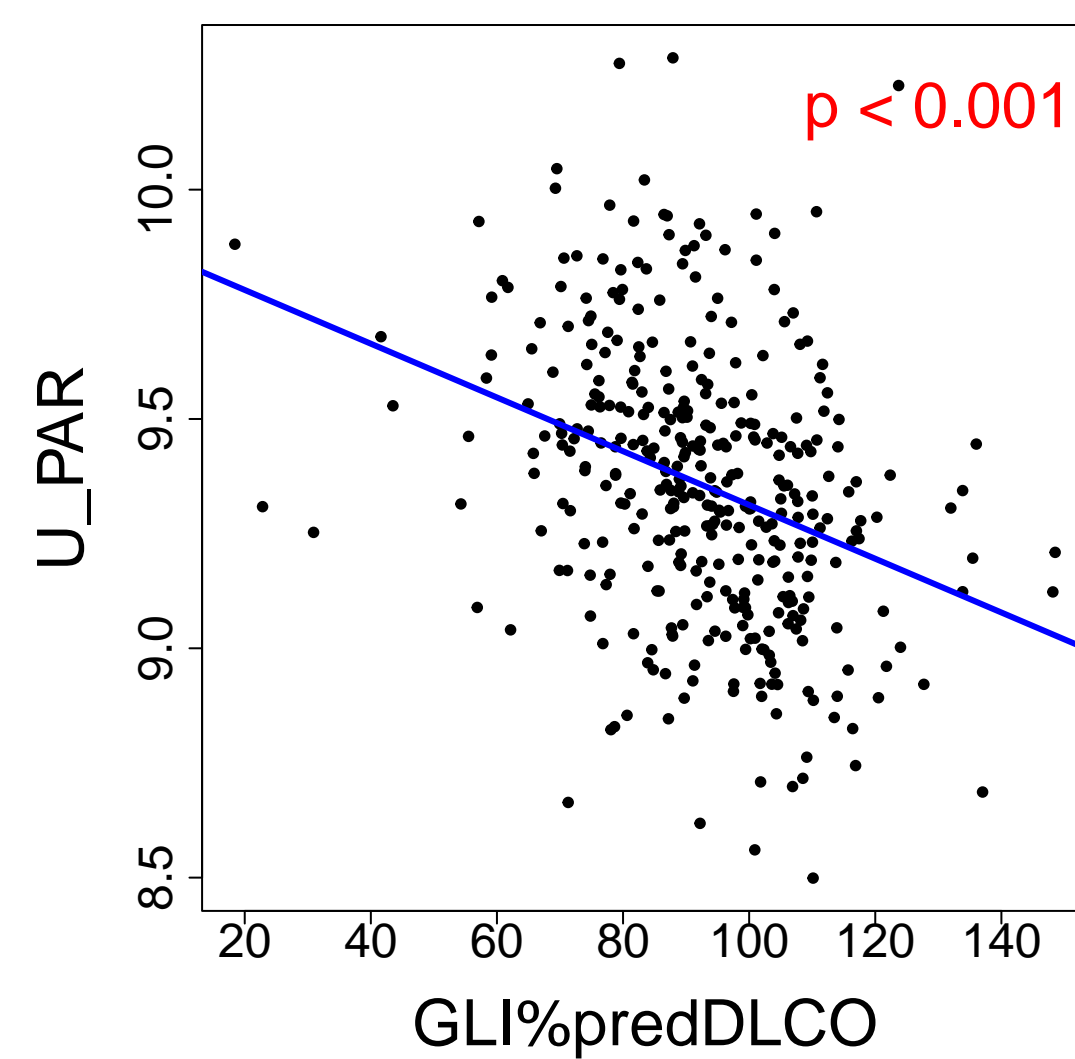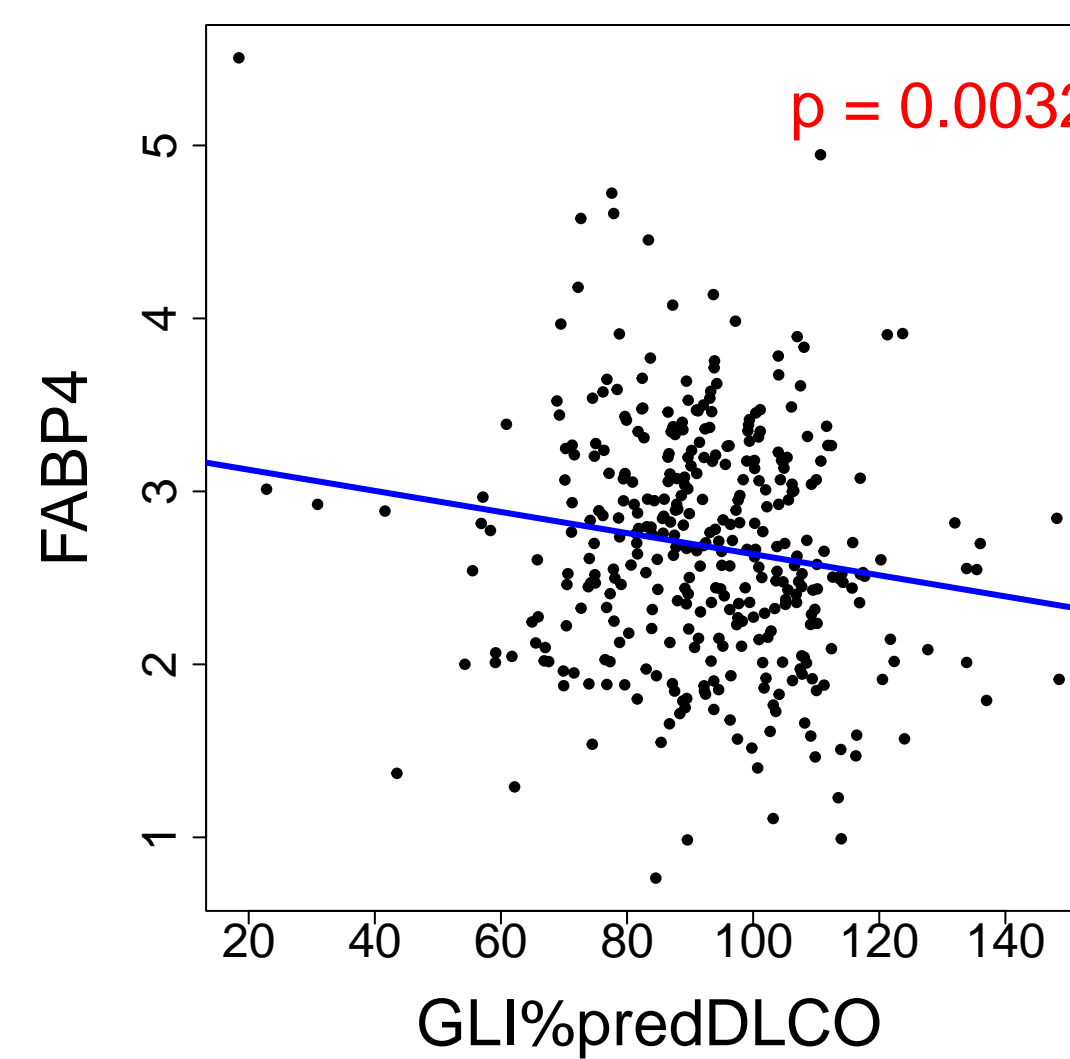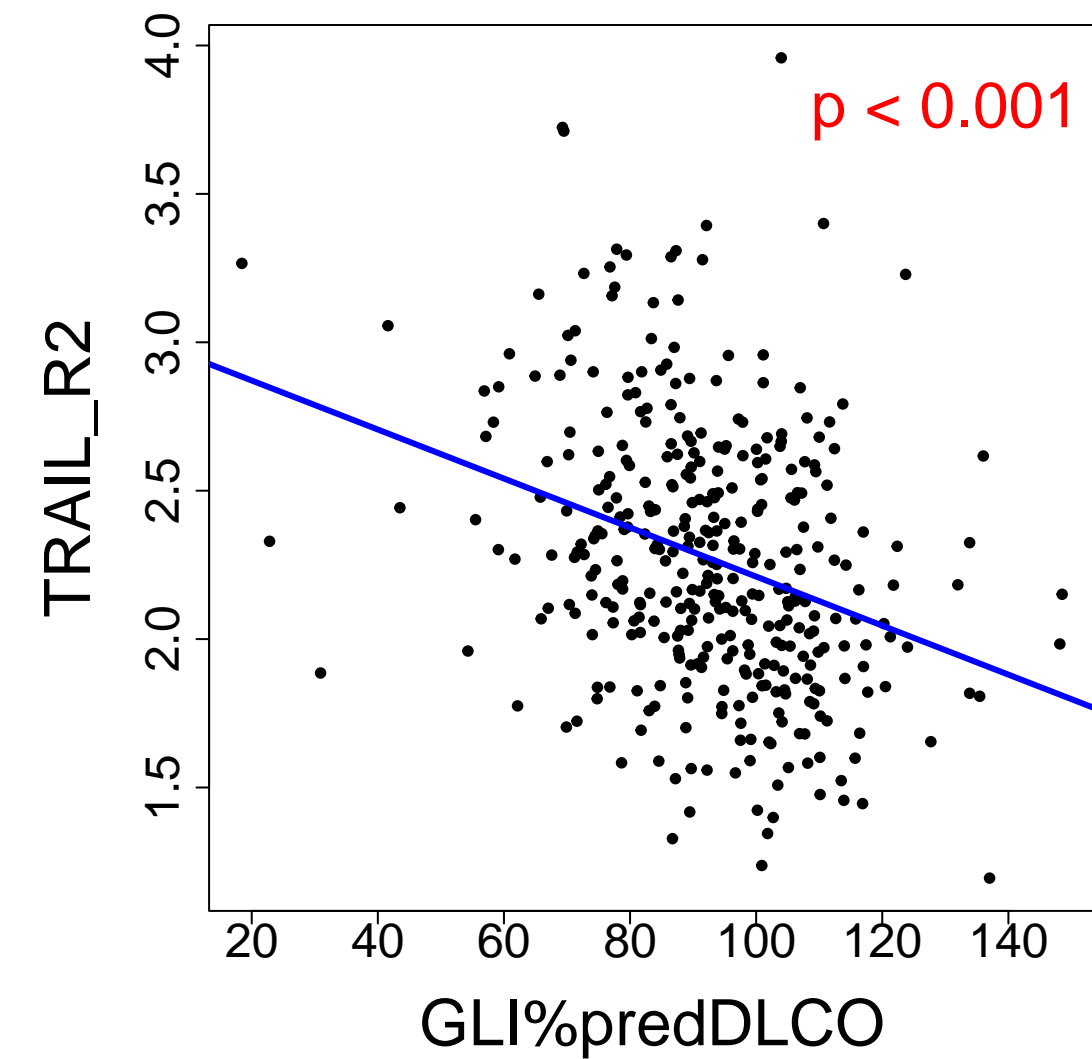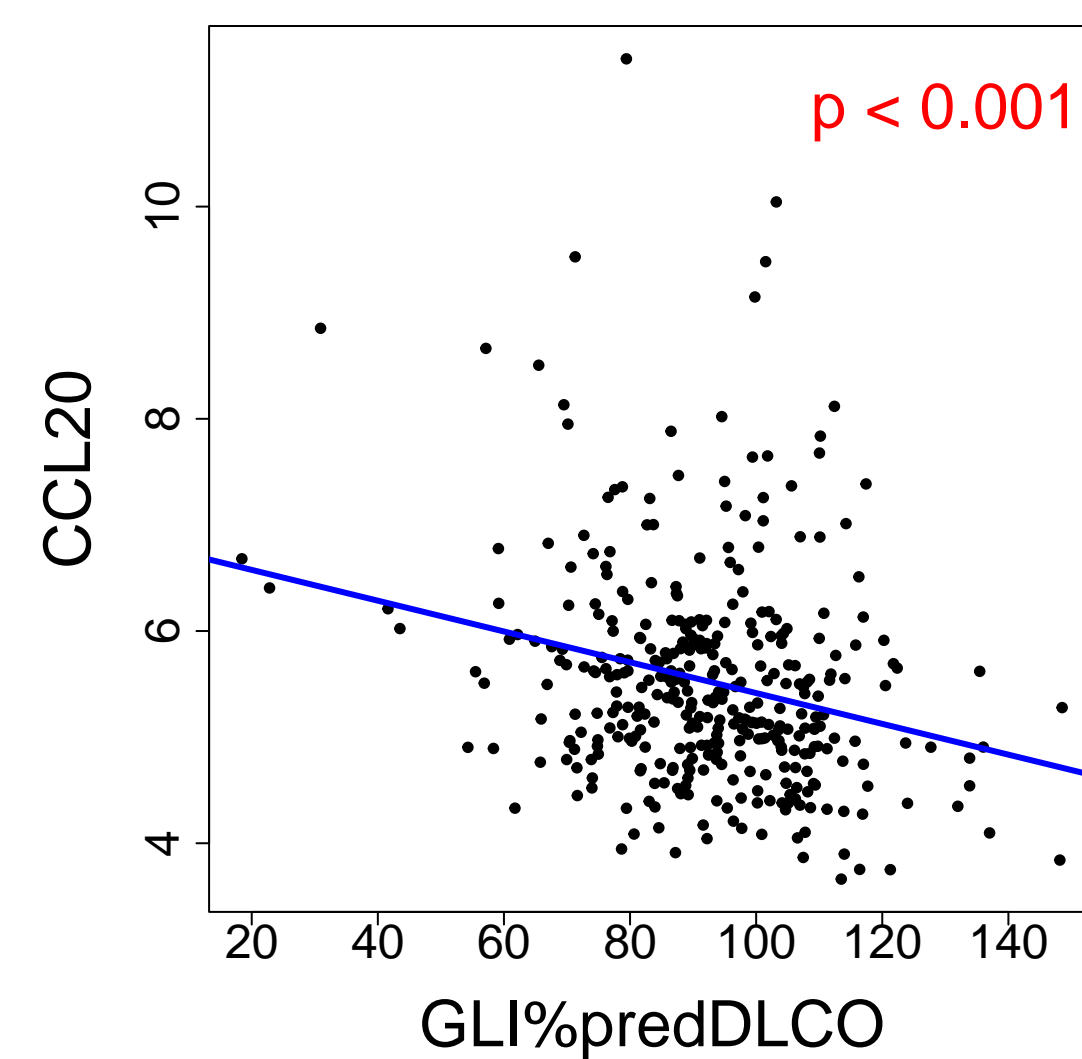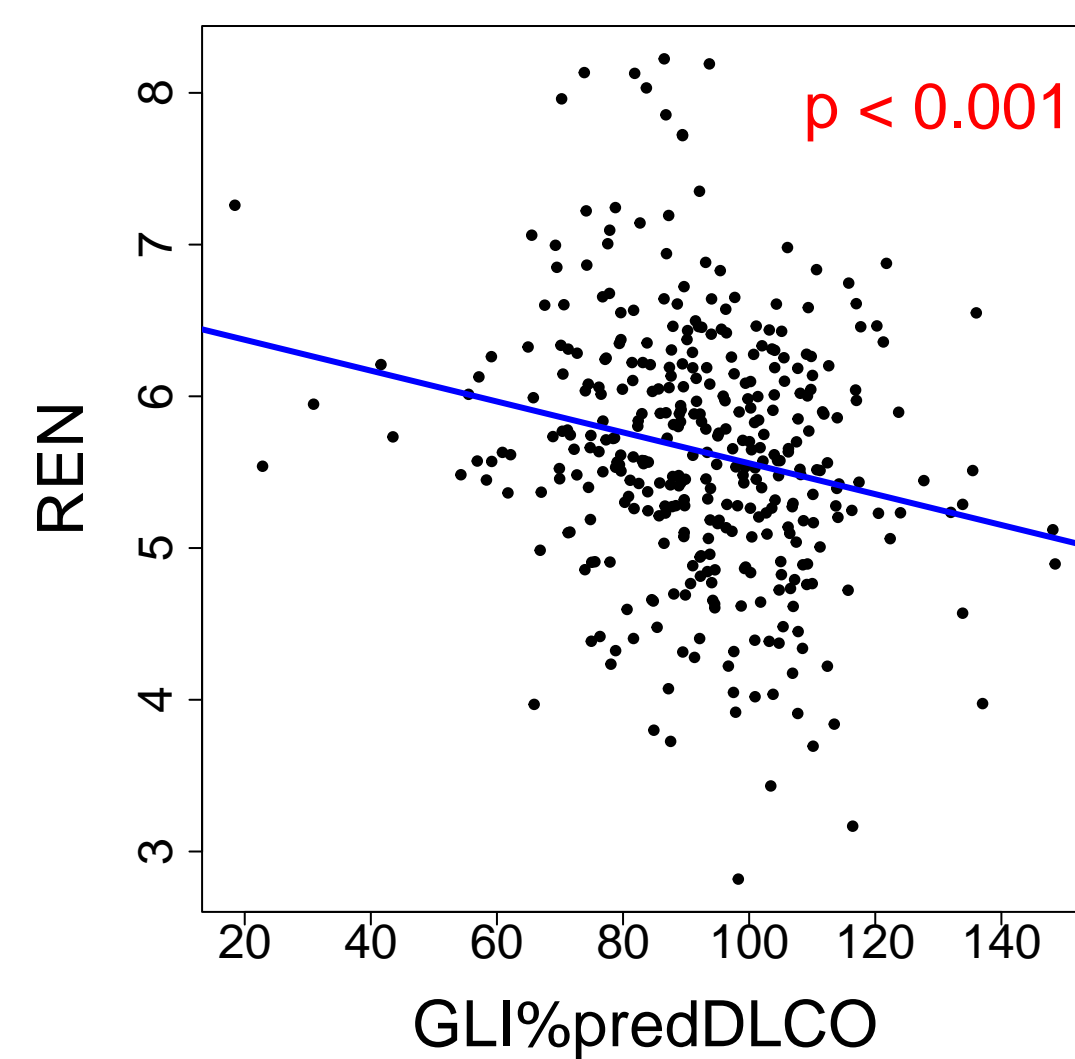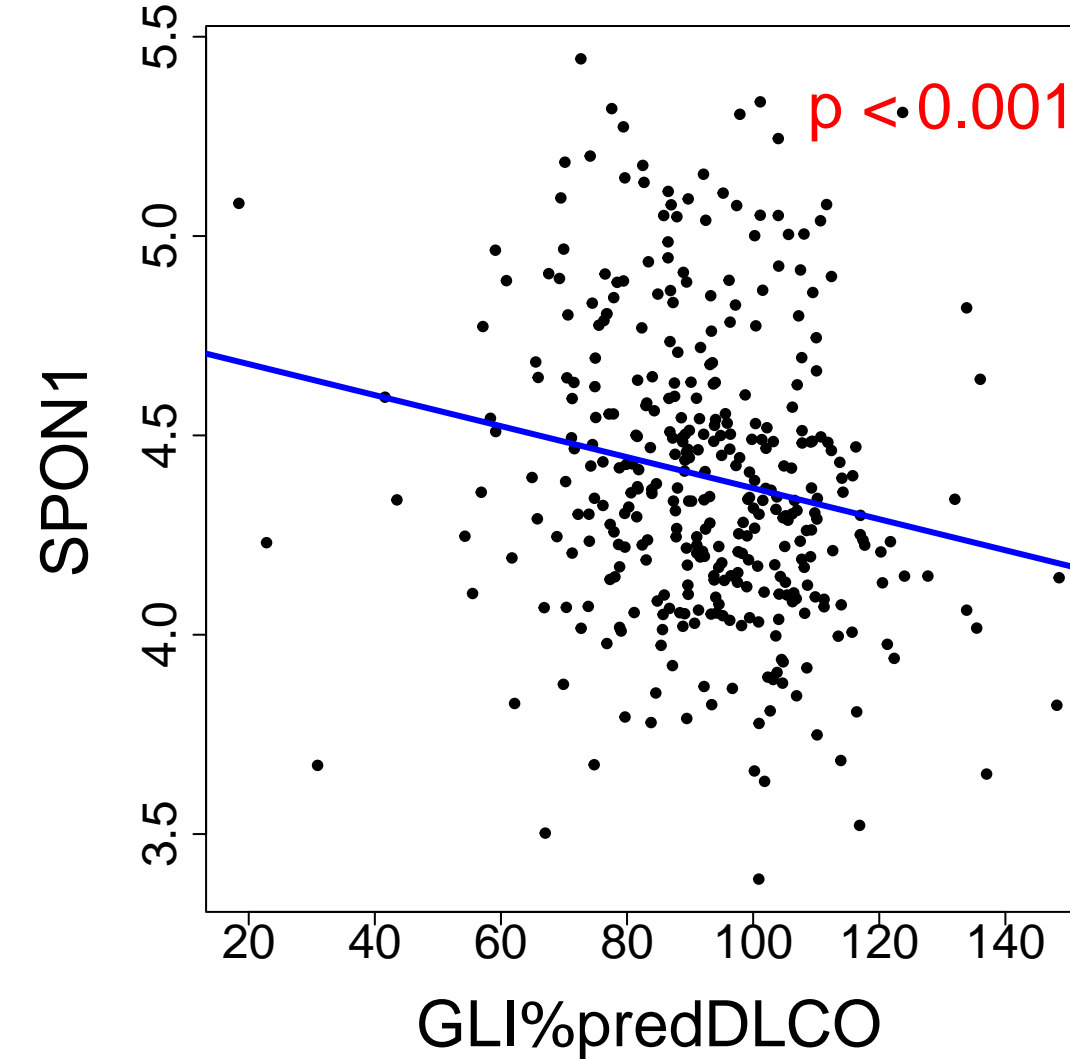

Supplement: Supplementary file 3 — Supplementary Material 3 [file 12014_2026_9584_MOESM3_ESM.pdf]
